# OXIDOSQUALENE CYCLASE 1 and 2 influence triterpene biosynthesis and defense in *Nicotiana attenuata*

**DOI:** 10.1093/plphys/kiad643

**Published:** 2023-12-15

**Authors:** Caiqiong Yang, Rayko Halitschke, Sarah E O'Connor

**Affiliations:** Department of Molecular Ecology, Max Planck Institute for Chemical Ecology, Hans-Knöll-Straße 8, Jena D-07745, Germany; Mass Spectrometry and Metabolomics, Max Planck Institute for Chemical Ecology, Hans-Knöll-Straße 8, Jena D-07745, Germany; Department of Natural Product Biosynthesis, Max Planck Institute for Chemical Ecology, Hans-Knöll-Straße 8, Jena D-07745, Germany

## Abstract

Triterpenes are a class of bioactive compounds with diverse biological functions, playing pivotal roles in plant defense against biotic stressors. Oxidosqualene cyclases (OSCs) serve as gatekeepers in the biosynthesis of triterpenes. In this study, we utilized a *Nicotiana benthamiana* heterologous expression system to characterize NaOSC1 from *Nicotiana attenuata* as a multifunctional enzyme capable of synthesizing lupeol, dammarenediol II, 3-alpha,20-lupanediol, and 7 other triterpene scaffolds. We also demonstrated that NaOSC2 is, in contrast, a selective enzyme, producing only the β-amyrin scaffold. Through virus-induced gene silencing and in vitro toxicity assays, we elucidated the roles of NaOSC1 and NaOSC2 in the defense of *N. attenuata* against *Manduca sexta* larvae. Metabolomic and feature-based molecular network analyses of leaves with silenced *NaOSC1* and *NaOSC2* unveiled 3 potential triterpene glycoside metabolite clusters. Interestingly, features identified as triterpenes within these clusters displayed a significant negative correlation with larval mass. Our study highlights the pivotal roles of NaOSC1 and NaOSC2 from *N. attenuata* in the initial steps of triterpene biosynthesis, subsequently influencing defense against *M. sexta* through the modulation of downstream triterpene glycoside compounds.

## Teaser

Two oxidosqualene cyclases mediate triterpene biosynthesis, which contributes to *Nicotiana attenuata* defense against *Manduca sexta* larvae.

## Introduction

Natural sources have yielded a remarkable diversity of thousands of distinct triterpenoids, featuring more than 100 structural variations ranging from simple acyclic to complex hexacyclic forms (Thimmappa et al. 2014; Hill and Connolly 2017). The first committed step of triterpenoid biosynthesis is the cyclization of 2,3-oxidosqualene, a pivotal step catalyzed by oxidosqualene cyclase (OSC) enzymes in plants (Vranova et al. 2013; Noushahi et al. 2022). In this enzymatic mechanism, the 2,3-oxidosqualene substrate adopts a chair–chair–chair conformation, leading to the formation of the dammarenyl carbocation intermediate before cyclization, giving rise to various triterpenoid types such as ursane, oleanane, lupane, and other diverse skeletal structures (Phillips et al. 2006; Thimmappa et al. 2014). Conversely, in sterol biosynthesis, different OSCs are involved, where 2,3-oxidosqualene assumes a chair–boat–chair conformation, resulting in the formation of the protosteryl cation intermediate (Kolesnikova et al. 2006; Suzuki et al. 2006). Many plant OSCs have been cloned and characterized using yeast or *Nicotiana benthamiana* heterologous expression systems. A subset of OSCs has been shown to convert 2,3-oxidosqualene to triterpenoids. Most of these functionally characterized OSCs catalyze oxidosqualene into β-amyrin, α-amyrin, or lupeol; only a few OSCs that catalyze the formation of other triterpene products have been characterized. Currently, the majority of characterized plant OSCs primarily produce a single class of triterpenoid products. However, some OSCs are multifunctional (Kawano et al. 2002; Wang et al. 2011; Misra et al. 2014; Moses et al. 2015; Andre et al. 2016; Srisawat et al. 2019). The model plant Arabidopsis (*Arabidopsis thaliana*) provides a glimpse into the remarkable biochemical diversity of OSCs. The *A. thaliana* genome harbors 13 OSC genes (Fazio et al. 2004), including 6 with specific cyclization functions and 6 multifunctional OSCs, which produce mixtures of products including cycloartenol, thalianol, marneral, arabidiol, lanosterol, β-amyrin, lupeol, 3-alpha,20-lupanediol, germanol, and camelliol C (Herrera et al. 1998; Kushiro et al. 2000a; Husselstein–Muller et al. 2001; Ebizuka et al. 2003; Suzuki et al. 2006; Xiang et al. 2006; Kolesnikova et al. 2007; Lodeiro et al. 2007; Shibuya et al. 2009).

Triterpenes exhibit diverse biological activities, including antibacterial, antifungal, antiparasitic, insecticidal, and antifeedant qualities (Morrissey and Osbourn 1999; Tian et al. 2021; Kuzminac et al. 2023). Azadirachtin, a tetranortriterpene isolated from the Indian neem tree *Azadirachta indica*, possesses potent insect antifeedant properties along with growth regulatory and reproductive effects (Arnason et al. 1985; Dawkar et al. 2019). At present, certain studies have elucidated the initial biosynthesis of these intricate triterpenoid compounds (Hodgson et al. 2019). Quassinoids, another group of well-known nortriterpenes, are also recognized for their insecticidal properties (Curcino Vieira and Braz-Filho 2006; He et al. 2020). Betulinic acid, ursolic acid, and their derivatives exhibit larvicidal activity against *Aedes aegypti* larvae, with the hydroxyl group playing an essential role in their larvicidal potential (Silva et al. 2016). β-Amyrin exhibited concentration-dependent antifeedant activity against *Spodoptera litura*, which increases the larval and pupal duration and mortality (Kannan et al. 2013). Oleanolic acid, maslinic acid, and their derivatives isolated from *Junellia aspera* (Verbenaceae) exhibit insecticidal or antifeedant activity against *Sitophilus oryzae* (Pungitore et al. 2005). Apart from these simple triterpenes, glycosylated triterpenoid compounds exhibit strong repellent or deterrent activity against herbivores and have shown insecticidal effects on aphids, beetles, weevils, leafhoppers, worms, and moths (Stevenson et al. 2009; Nielsen et al. 2010; D’Addabbo et al. 2011; Da Silva et al. 2012; De Geyter et al. 2012).

*Nicotiana attenuata*, an annual native wild tobacco indigenous to the Great Basin Desert in the Southwestern United States, serves as a valuable model plant for investigating plant–herbivore interactions. Extensive research has revealed the ecological functions of various specialized metabolites, including acyl sugars, nicotine, diterpene glycosides, and phenolamines, in the context of plant–herbivore interactions (Weinhold and Baldwin 2011; Gaquerel et al. 2014; Kumar et al. 2014). However, the composition of the triterpene reservoir of *N. attenuata*, along with the associated ecological functions, remains undiscovered. While certain *Nicotiana* species have been reported to contain lupane- and oleane-type triterpenes (Popova et al. 2018, 2019, 2020), it is important to note that the content and diversity of these compounds exhibit substantial variation across different species.

Furthermore, to the best of our knowledge, no OSCs have been characterized in the *Nicotiana* genus, and none have been identified in *N. attenuata*. The primary objective of this study is to identify and characterize the OSCs that produce the triterpene scaffolds of the important ecological model plant *N. attenuata*. Here, we identify and biochemically characterize 2 key OSCs of *N. attenuata*. We then demonstrate the roles that these cyclization enzymes play in the plant’s defense against herbivores. We employed a homology-based approach to identify candidate OSCs in *N. attenuata* and functionally characterized them using heterologous expression in the *N. benthamiana* system. We employed virus-induced gene silencing (VIGS) to silence these NaOSCs and investigated the resulting impact on the interaction with *Manduca sexta*, a native herbivore of *N. attenuata*. In addition, we conducted an in vitro bioassay of NaOSC enzyme products for their toxicity against *M. sexta* larvae. In summary, the identification of 2 key OSCs in *N. attenuata* allowed us to demonstrate the important role of triterpenes in the defense of this important plant.

## Results

### Identification and sequence analysis of OSCs from *N. attenuata*

**Figure 1.**
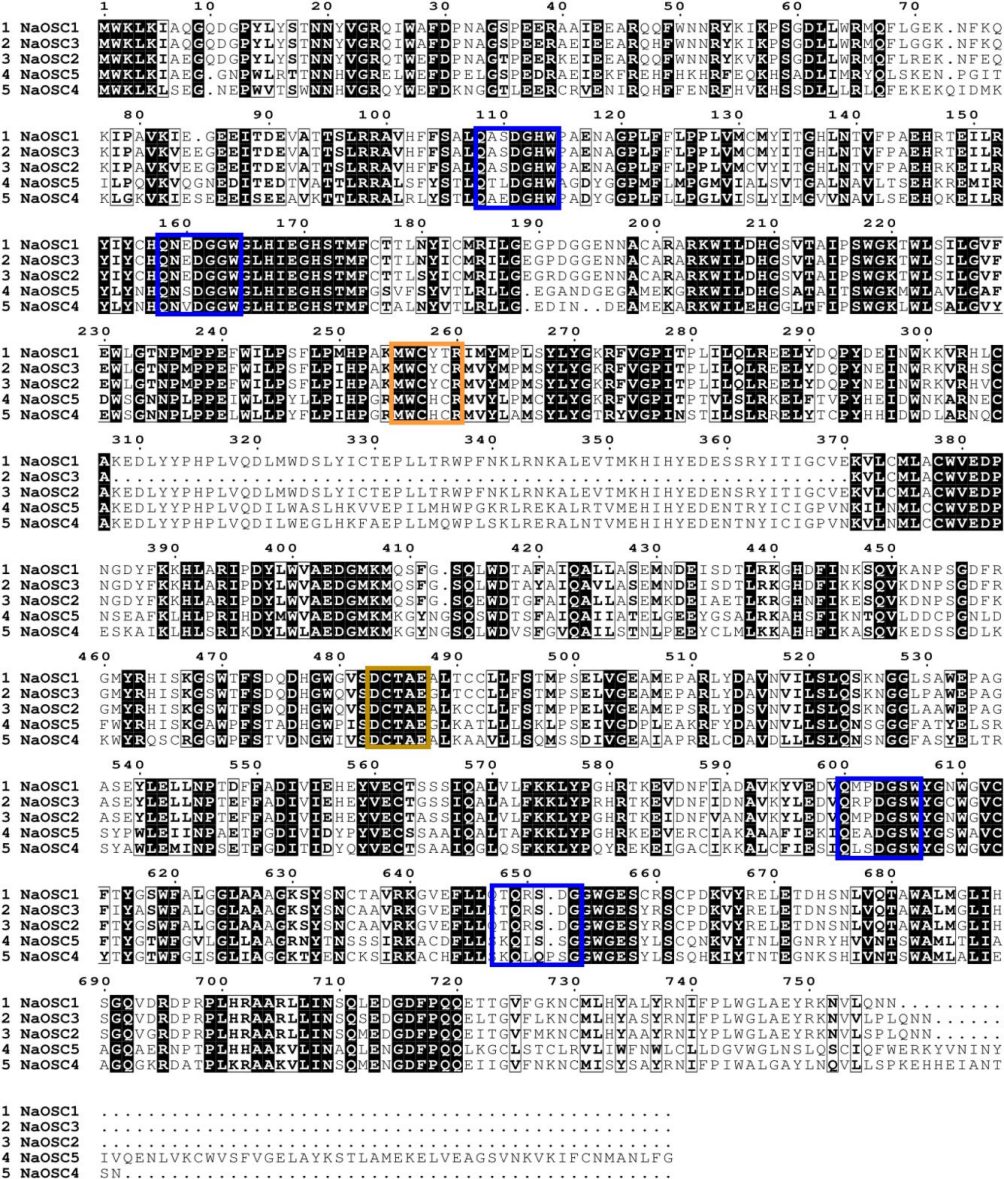
Multiple alignments of amino acid sequences of *NaOSCs* from *N. attenuata*. In lines 2, 3, 8, and 9 of Fig. 1, the box marks are the QW motifs. In line 4 of Fig. 1 the box indicates the MWCYCR motif, and in line 7, the box marks the DCTEA motif.

**Figure 2.**
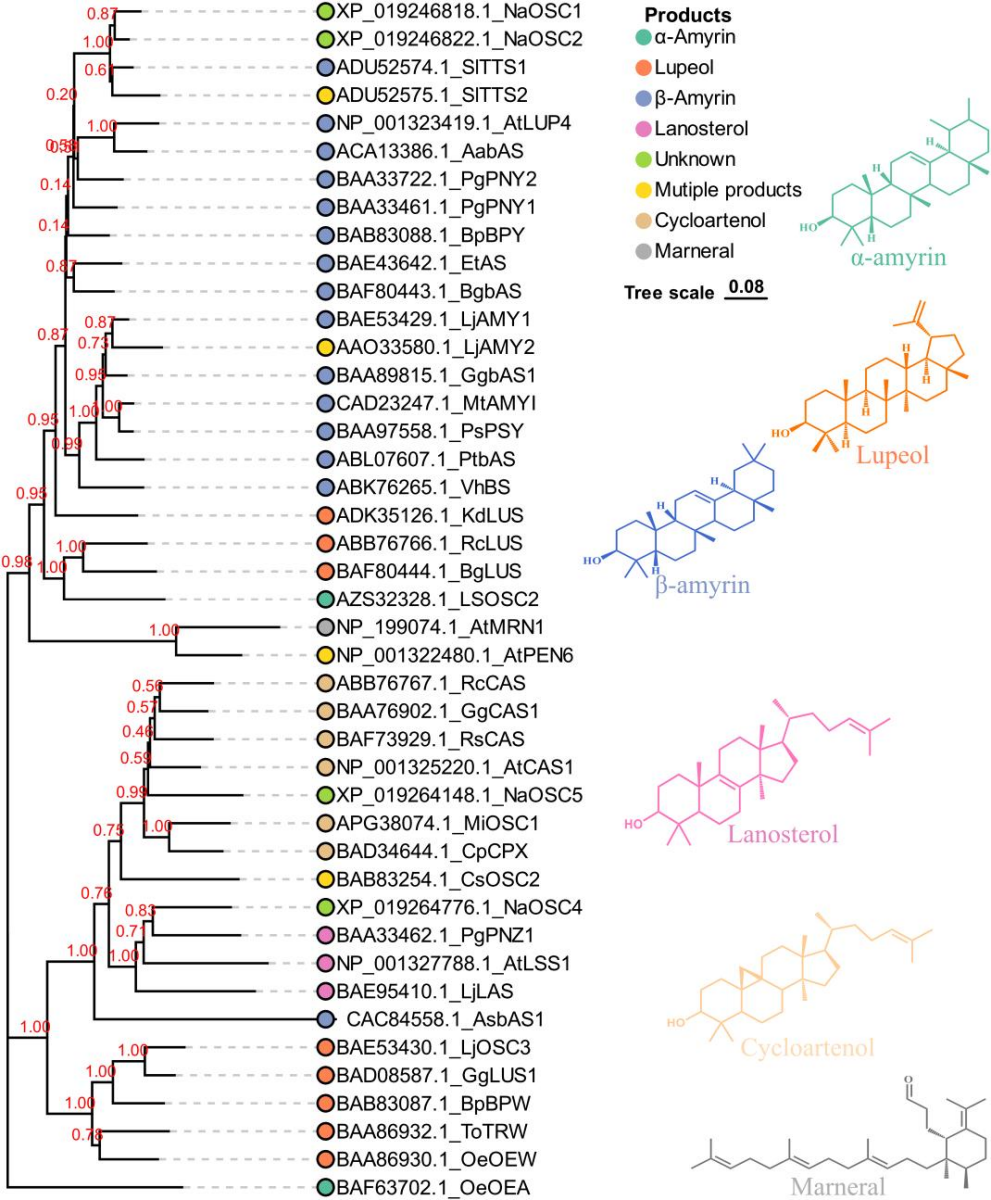
Phylogenetic analysis of OSCs in *N. attenuata* and comparative species. OSCs used for phylogenetic analysis in this study were obtained from the literature list provided in Supplemental Table S1. Amino acid sequences of OSCs were aligned using ClustalW with default parameters, as implemented in the MEGA 11 software. A neighbor-joining tree was constructed and subjected to bootstrapping analysis (1,000 iterations). Distances were computed using the Jones-Toyler-Thornton matrix–based method. The scale bar shows the branch length representing 0.05 amino acid substitutions per site. The numerals along the branches represent bootstrap values, denoting the statistical support for the tree’s topology in a phylogenetic context. Different colored symbols indicate OSCs with distinct products.

Based on the hidden Markov model and BlastP results, a total of 6 candidate OSCs, designated as *NaOSC1* to *NaOSC6*, were predicted to be present in the *N. attenuata* genome. Among these candidate genes, *NaOSC6* exhibits a notably shorter DNA length, measuring fewer than 500 base pairs, which is substantially smaller than the other NaOSCs (Supplemental Table S1). Additionally, sequence analysis revealed a 62-base-pair gap within the amino acid sequence of NaOSC3, spanning positions 308 to 370, distinguishing it from the other NaOSCs (Fig. 1). Consequently, these 2 genes were excluded from further functional characterization. Consistent with other OSCs, all NaOSCs contain the highly conserved DCTAE motif (Fig. 1; Supplemental Fig. S1), implicated in substrate binding (Siedenburg and Jendrossek 2011), along with the QW motifs (Fig. 1; Supplemental Fig. S1) that are suggested to stabilize the carbocation intermediate during cyclization (Kushiro et al. 2000b). NaOSC2 also includes the conserved MWCYCR motif, commonly found in previously identified β-amyrin synthases (Kushiro et al. 2000a). In NaOSC1, the penultimate cysteine of the MWCYCR motif is replaced by serine (Fig. 1; Supplemental Fig. S1). NaOSC4 and NaOSC5 contain the MWCHCR motif, which facilitates the formation of the protosteryl cation intermediate, that in turn leads to the formation of the sterol backbones (Corey et al. 1997). Phylogenetic analysis of candidate OSCs from *N. attenuata*, alongside previously reported OSCs (Supplemental Table S2), reveals that NaOSC1 and NaOSC2 share high sequence identity and similarity with SlTTS1 and SlTTS2 from *Solanum lycopersicum*, clustering within the evolutionary branch containing β-amyrin synthases (Fig. 2). NaOSC4 and NaOSC5 cluster with OSCs possessing oxidizing activity in lanosterol synthases and cycloartenol synthases (Fig. 2).

### *NaOSC1* and *NaOSC2* contribute to triterpene diversity in *N. attenuata*

**Figure 3.**
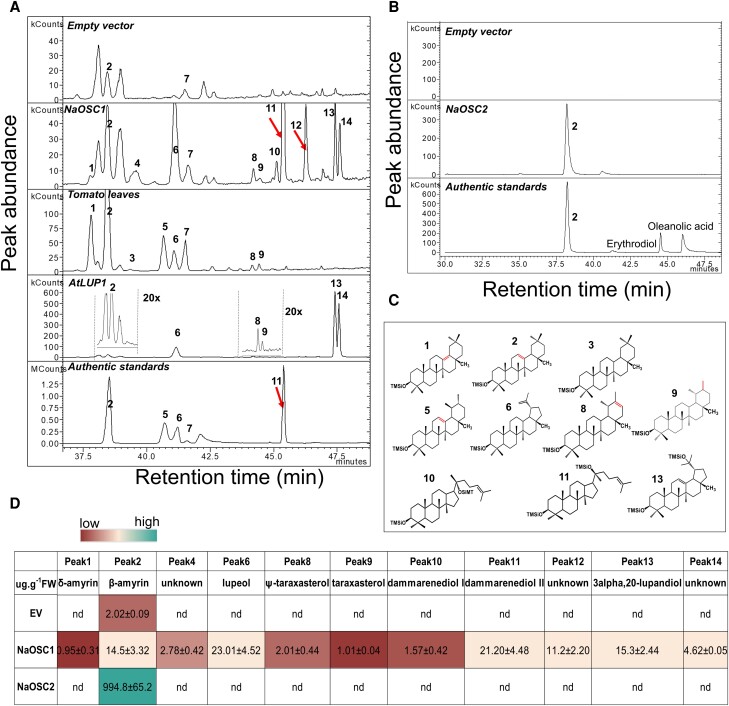
Functional expression of *NaOSC1* and *NaOSC2* in *N. benthamiana* and identification of the enzyme products. **A)** GC-MS extracted ion chromatograms (189, 199, 131, 203, and 218) of *NaOSC1*-transiently expressed *N. benthamiana* leaf. The “20x” in **A)** signifies a 20-fold zoom out of the view. KCounts and MCounts indicate peak intensity units as kilo counts and mega counts, respectively. **B)** GC-MS extracted ion chromatograms (189, 203, and 218) of *NaOSC2*-transiently expressed *N. benthamiana* leaf. **C)** Structures of triterpene products from *NaOSC1 and NaOSC2*. **D)** The relative content of triterpene products (mean ± Se, *n* = 3; the color filling of the squares indicates the level of product content; nd, no peak is detected; the relative content was calculated based on the internal standard, α-amyrin). Numeric labels represent distinct triterpene compounds: 1, δ-amyrin; 2, β-amyrin; 3, γ-amyrin; 4, unknown; 5, α-amyrin (internal standard); 6, lupeol; 7, cycloartenol; 8, ψ-taraxasterol; 9, taraxasterol; 10, dammarenediol I; 11, dammarenediol II; 12, unknown; 13, 3-alpha,20-lupandiol; 14, unknown.

To biochemically characterize the putative OSCs from *N. attenuata*, heterologous expression of the corresponding genes was performed in *N. benthamiana* plants. The full-length complementary DNAs (cDNAs) for *NaOSC1*, *NaOSC2*, *NaOSC4*, and *NaOSC5* were cloned into a 3Ω1 vector (Cárdenas et al. 2019) and subsequently transformed into *Agrobacterium tumefaciens*. *A. tumefaciens* cells were infiltrated into *N. benthamiana* leaves and harvested after 5 d for triterpene analysis. Leaves infiltrated with empty 3Ω1 vector served as the negative control. In leaves overexpressing NaOSC1, the peak corresponding to the β-amyrin standard exhibited a 7-fold increase compared to the negative control, confirming the production of β-amyrin by NaOSC1 (Fig. 3, A, C, and D). Furthermore, we observed 10 additional peaks in the GC chromatograms of leaves transiently expressing NaOSC1. We observed peaks with retention time and Electron Ionization (EI) mass spectrum matching lupeol and dammarenediol II standards (Fig. 3A; Supplemental Fig. S2). An additional peak (Peak 10) was hypothesized to be dammarenediol I, an isomer of dammarenediol II (Fig. 3A; Supplemental Fig. S2). Among the other unknown peaks, Peaks 1, 8, and 9 have highly similar EI mass spectra to δ-amyrin, ψ-taraxasterol, and taraxasterol, respectively (Fig. 3, A and C;Supplemental Fig. S2), compounds reported in *S. lycopersicum* (Wang et al. 2011; Shan et al. 2015). Additionally, Peak 13 closely resembles the EI mass spectrum of 3-alpha,20-lupanediol (Fig. 3, A and C;Supplemental Fig. S2), a major product of AtLUP1 in *A. thaliana* (Herrera et al. 1998; Segura et al. 2000). Peak 14, which is not consistently present in all samples, is likely a derivative of 3-alpha,20-lupanediol. Hence, through a comparative analysis of retention times and EI mass spectra of triterpenes derived from NaOSC1, AtLUP1, and *S. lycopersicum* leaves, we propose that NaOSC1 is, in addition to the production of β-amyrin, also involved in the biosynthesis of δ-amyrin, ψ-taraxasterol, taraxasterol, and 3-alpha,20-lupanediol (Fig. 3A; Supplemental Fig. S2). An additional remaining 3 compounds synthesized by NaOSC1 could not be conclusively identified. Conversely, NaOSC2-expressing leaves produced solely β-amyrin (Fig. 3B), with yields approximately 80 times higher than those produced by leaves overexpressing NaOSC1 (Fig. 3D). Leaves transiently expressing NaOSC4 or NaOSC5 did not yield any additional products compared to the negative control with the empty vector (Supplemental Fig. S3). In summary, NaOSC1 exhibits low product specificity, producing β-amyrin, lupeol, dammarenediol II, and 3-alpha,20-lupanediol as major products, while NaOSC2 is a highly specific enzyme, exclusively synthesizing β-amyrin.

### Spatiotemporal accumulation of enzyme products of NaOSC1 and NaOSC2 in *N. attenuata*

**Figure 4.**
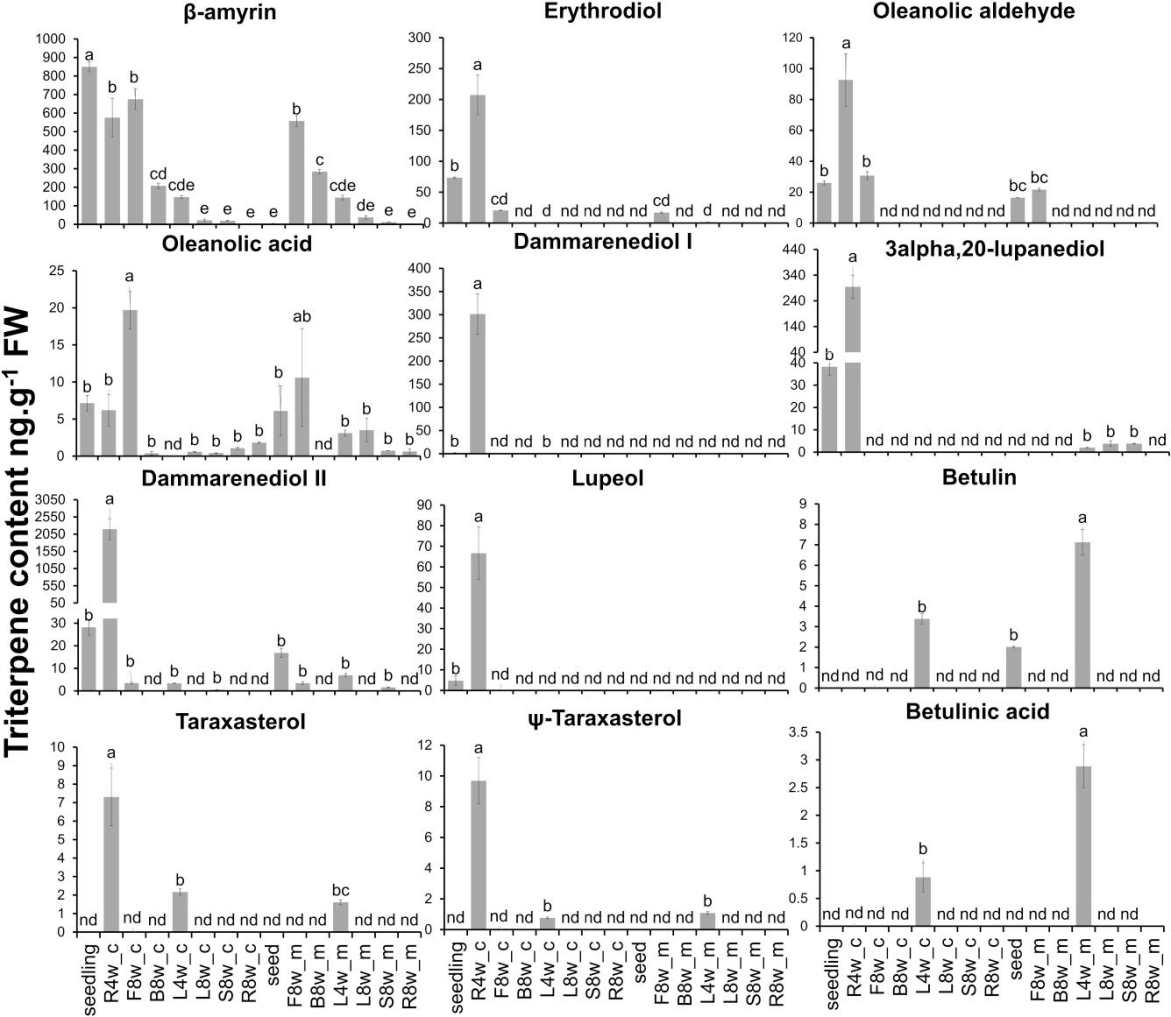
Temporal and spatial accumulation of triterpenes in *N. attenuata*. R4w, 4-wk-old roots; F8w, 8-wk-old flowers; B8w, 8-wk-old flower buds; L4w, 4-wk-old leaves; L8w, 8-wk-old leaves; S8w, 8-wk-old stems; R8w, 8-wk-old roots; _c, plants treated with lanolin without methyl jasmonate for 72 h; _m, plants treated with 250 *μ*g of methyl jasmonate in lanolin for 72 h. Data are shown in mean ± Se, *n* = 4, 1-way ANOVA, Tukey’s test; different lowercase letters indicate significant differences among levels of triterpene content (*P* ≤ 0.05). nd, no triterpenes were detected.

We next assessed the triterpenoid profile in *N. attenuata*. We profiled seeds, 14-d-old seedlings, 4-wk-old plants, and 8-wk-old *N. attenuata* plants, as well as root, stem, leaf, flower bud, and fully open flower *N. attenuata* samples. β-Amyrin, 3-alpha,20-lupanediol, lupeol, ψ-taraxasterol, taraxasterol, dammarenediol II, and their oxidized derivatives erythrodiol, betulin, oleanolic acid, and oleanolic aldehyde were detected (Fig. 4; Supplemental Fig. S4). We observed pronounced temporal and tissue-specific accumulation of these triterpenes. The shared product of NaOSC1 and NaOSC2, β-amyrin, was present in nearly all examined plant tissues, with the highest accumulation observed in the 14-d-old seedlings, roots of 4-wk-old plants, and flowers, reaching 849, 575, and 616 ng per gram of fresh weight (FW) plant tissue, respectively (Fig. 4). The 2 major products of NaOSC1, 3-alpha,20-lupanediol and dammarenediol II, exhibited the highest accumulation in 14-d-old seedlings and roots of 4-wk-old plants, with levels reaching 2,193 and 294 ng g^−1^ FW, respectively. Furthermore, these compounds were also detected in leaves and stems following methyl jasmonate induction, albeit at a low level. Other products of NaOSC1, such as lupeol, ψ-taraxasterol, and taraxasterol, were only detected in 14-d-old seedlings, 4-wk-old plant leaves, or roots of 4-wk-old plants, with concentrations ranging from 7 to 60 ng g^−1^ FW (Fig. 4). In addition to the simple triterpene scaffolds produced by NaOSC1 and NaOSC2, derivatives of β-amyrin, including erythrodiol (73 to 207 ng g^−1^ FW), oleanolic aldehyde (30 to 92 ng g^−1^ FW), and oleanolic acid (6 ng g^−1^ FW), as well as derivatives of lupeol, betulin, and betulinic acid, were detected in leaves, seedlings, or roots of 4-wk-old plants. Some of these derivatives, such as oleanolic acid, erythrodiol, betulin, and betulinic acid, were induced in leaves or stems following methyl jasmonate treatment. Additionally, oleanolic acid, erythrodiol, and oleanolic aldehyde were also detected in flowers (Fig. 4). The metabolic profile of the NaOSC1 and NaOSC2 products leads to a wide variety of triterpene derivatives in all *N. attenuata* tissues.

### Tissue-specific and inducible expression patterns of *NaOSC1* and *NaOSC2*

**Figure 5.**
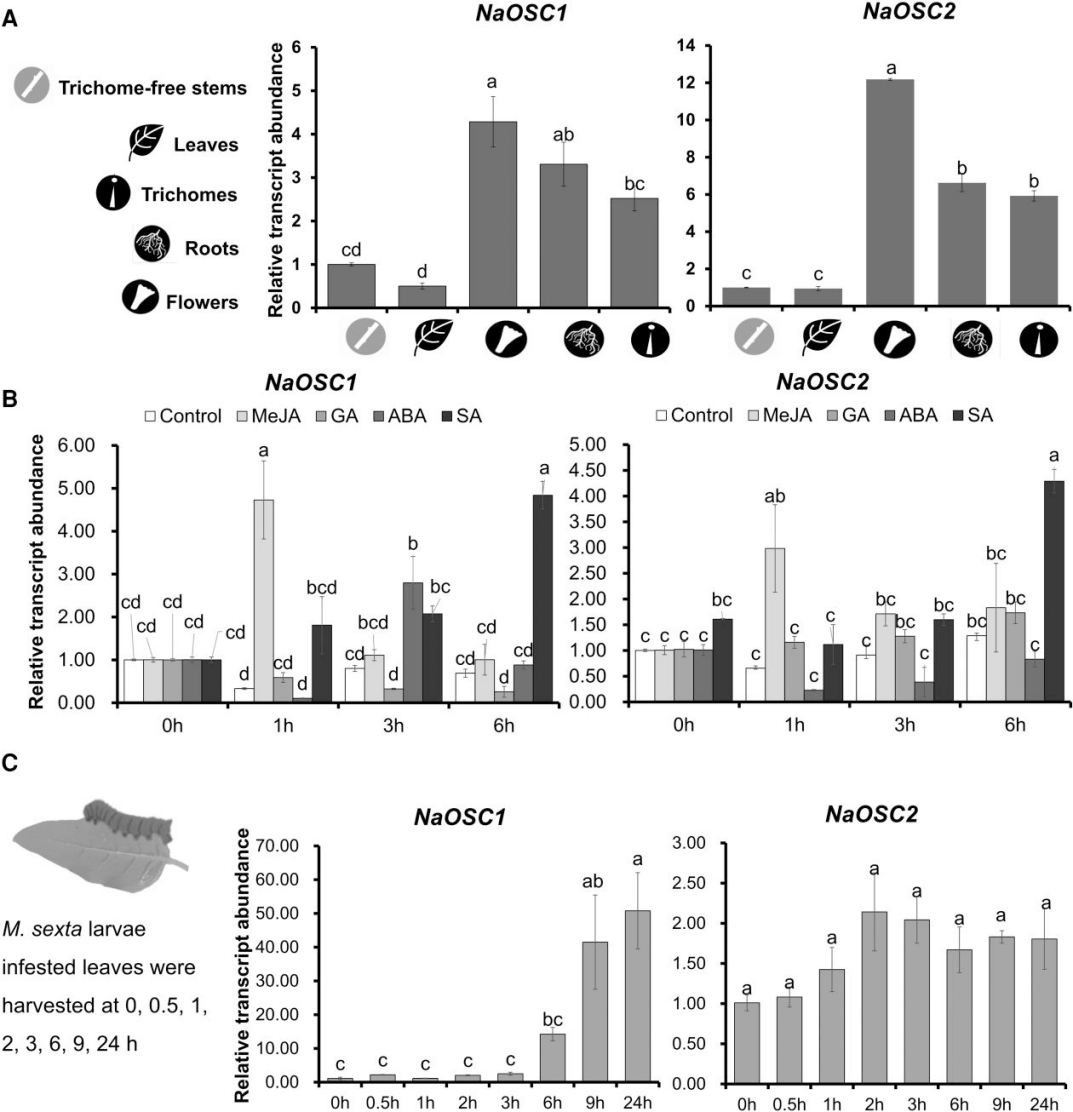
Expression patterns of *NaOSC1* and *NaOSC2*. **A)** Tissue-specific expression of *NaOSC1* and *NaOSC2*. Plant tissues were collected from 8-wk-old plants (initiation of flowering). **B)** Expression specificity of *NaOSC1* and *NaOSC2* in response to phytohormone induction. Phytohormone induction was performed on 14-d-old seedlings. GA, gibberellin A_3_ (100 *μ*M); MeJA, methyl jasmonate (100 *μ*M); ABA, abscisic acid (100 *μ*M); SA, salicylic acid (100 *μ*M). **C)** Response of *NaOSC1* and *NaOSC2* to *M. sexta* larval infestation. Infestation of larvae conducted on 4-wk-old plants. Results of ANOVAs and Tukey's test are used in statistical analysis and shown as mean ± Se, *n* = 3; different letters indicate statistically significant among levels of relative transcript abundance (*P* ≤ 0.05).

Reverse transcription quantitative PCR (RT-qPCR) analysis was performed to examine expression patterns of OSC genes in different plant tissues and under conditions of different hormone treatments (Fig. 5). *NaOSC1* and *NaOSC2* exhibit higher expression levels in flowers, roots, and trichomes compared to leaves and trichome-free stems, with the highest expression observed in flowers (Fig. 5A). The relative transcript abundance of *NaOSC1* and *NaOSC2* significantly increased within 1 h of methyl jasmonate treatment and within 3 to 6 h of salicylic acid treatment in 14-d-old *N. attenuata* seedlings. Additionally, a slight increase in the transcript abundance of *NaOSC1* was observed within 3 h of abscisic acid treatment (Fig. 5B). To investigate the responsiveness of *NaOSC1* and *NaOSC2* to the specialized herbivore of *N. attenuata*, we infested 4-wk-old plants with newly hatched *M. sexta* larvae and conducted RT-qPCR analyses at 0-, 0.5-, 1-, 2-, 3-, 6-, 9-, and 24-h postinfestation. The relative transcript abundance of *NaOSC1* in leaves increased significantly, ranging from 37- to 45-fold, during 9 to 24 h postlarval infestation, while *NaOSC2* exhibited no significant change in relative expression levels (Fig. 5C). Therefore, it appears that the multifunctional *NaOSC1* is induced in response to herbivore attack, whereas the expression of product-specific *NaOSC2* is not impacted by herbivores.

### *NaOSC1* and *NaOSC2* contribute to herbivore defense in *N. attenuata*

**Figure 6.**
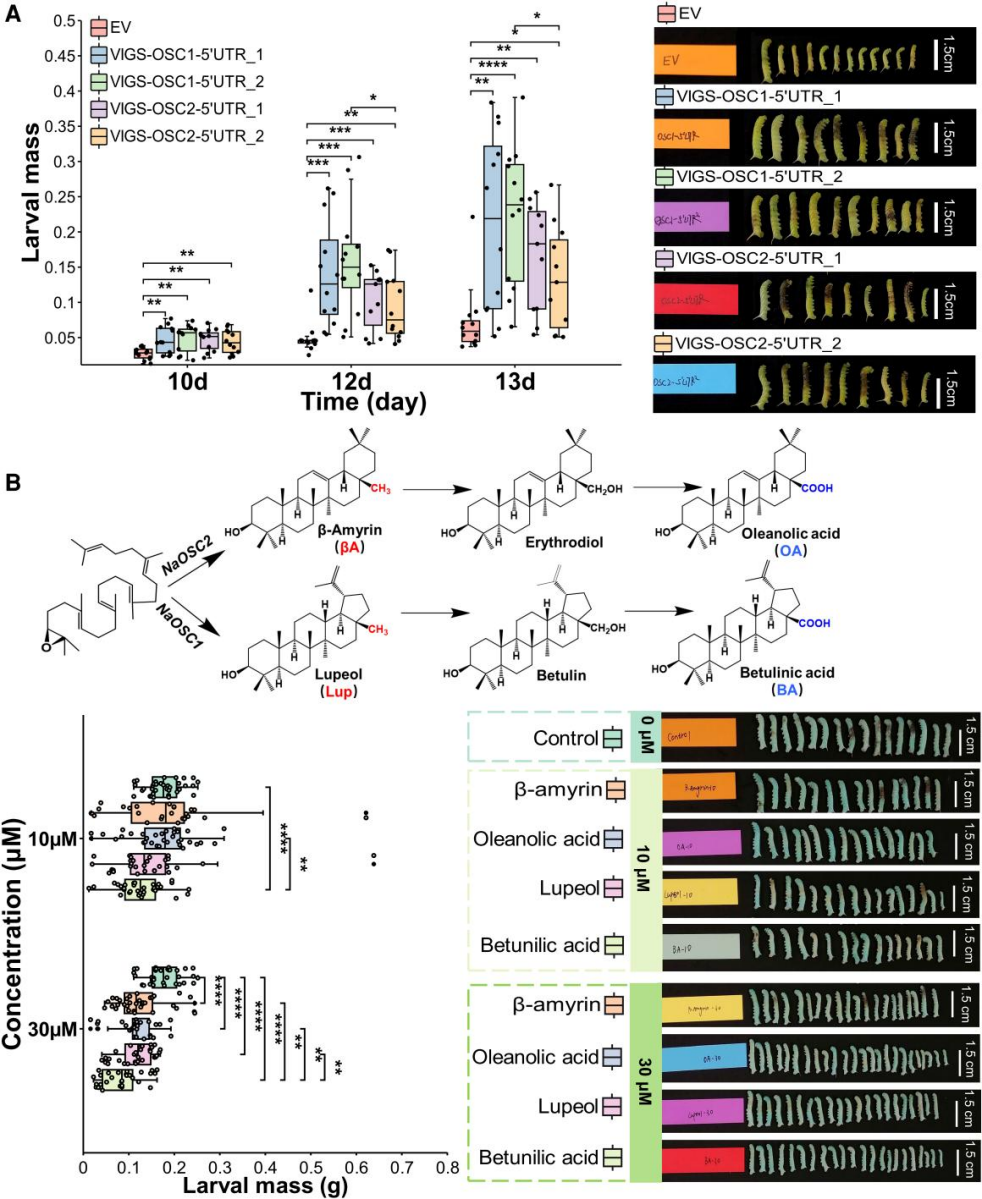
Impact of *NaOSC1* and *NaOSC2* on herbivore defense. **A)** The impact of VIGS-mediated silencing of *NaOSC1* and *NaOSC2* on the performance of *M. sexta* larvae. For each enzyme, 2 individual VIGS vectors were developed. Nonvirally symptomatic plants were removed 14 d post-*Agrobacterium* inoculation, maintaining 15 plants per vector. Larval treatments were initiated upon observing leaf whitening as the positive control (PDS). The *M. sexta* neonate is permitted unrestricted feeding on the plant for a duration of 13 d. **B)** Toxicity assessment of *NaOSC1* and *NaOSC2* products and derivatives on *M. sexta* larvae. For **A)** and **B)**, results of Wilcoxon's test is shown (*n* = 15 to 25, mean ± Se; *P ≤ 0.05; **P ≤ 0.01; ***P ≤ 0.001; ****P ≤ 0.0001). βA-10, 10 *μ*M β-amyrin; Lup-10, 10 *μ*M lupeol; BA-10, 10 *μ*M betulinic acid; OA-10, 10 *μ*M oleanolic acid; βA-30, 30 *μ*M β-amyrin; Lup-30, 30 *μ*M lupeol; BA-30, 30 *μ*M betulinic acid; OA-30, 30 *μ*M oleanolic acid. The central line within the box represents the median of the data. The upper and lower boundaries of the box denote the upper and lower quartiles of the data. The lines above and below the box, known as whiskers, signify the variability of the data (error bar). The whiskers’ length is set at 1.5 times the interquartile range. The points represent specific observations in the data set. The values beyond 1.5 times the interquartile range are considered as outliers.

To explore whether *NaOSC1* and *NaOSC2* contribute to herbivore defense in *N. attenuata*, we employed VIGS to silence *NaOSC1* and *NaOSC2* individually, which was achieved using a 250- to 300-bp segment located in the 5′UTR or 3′UTR. Compared to the negative control plants (empty vector [EV]), *NaOSC1* exhibited a reduction of 56% and 42% in *VIGS-OSC1-5′UTR_1* and *VIGS-OSC1-5′UTR_2* plants, respectively, while *NaOSC2* showed reductions of 30% and 35% in *VIGS-OSC2-5′UTR_1* and *VIGS-OSC2-5′UTR_2* plants, respectively (Supplemental Fig. S5). When the leaves of positive control plants (VIGS-PDS) showed the bleaching phenotype resulting from silencing of phytoene desaturase (at 14 d after inoculation), newly hatched *M. sexta* larvae were placed on the first fully expanded leaf of the plant stem, allowed to grow freely for 13 d, and then their larval mass was then recorded. Larvae that fed on *NaOSC1*-silenced plants (*VIGS-OSC1_5′UTR_1* and *VIGS-OSC1_5′UTR_2*) were 2.9 to 3.2 times higher in mass compared to larvae that fed on EV plants (Fig. 6A), while the larvae feeding on *NaOSC2-*silenced plants (*VIGS-OSC2_5′UTR_1* and *VIGS-OSC2_5′UTR_2*) gained 1.9 to 2.3 times higher masses compared to larvae that fed on EV plants (Fig. 6A). These results indicate that silencing of either *NaOSC1* or *NaOSC2* decreased the defenses of *N. attenuata* against *M. sexta* larvae.

For evaluation of the role of specific triterpene skeletons in defense against *M. sexta* larvae, we performed in vitro toxicity assays using their primary products and derived triterpene acids. Since many of the NaOSC1 products are not available in sufficient quantities, we restricted our assays to lupeol and its derived triterpene acid, betulinic acid. We also employed β-amyrin, which is produced by both NaOSC1 and NaOSC2, along with the downstream oleanolic acid for in vitro activity assays. Larval mass was significantly reduced in individuals consuming diets with 30 *μ*M of either β-amyrin, lupeol, oleanolic acid, or betulinic acid compared to the control group. Notably, larvae fed a diet containing 30 *μ*M betulinic acid exhibited a 46% reduction in mass compared to the control larvae (Fig. 6B). Larvae that were fed betulinic acid at lower concentrations (10 *µ*M) still showed significantly reduced mass compared to the control group fed a triterpene-free diet. The effect of betulinic acid at this lower concentration was stronger than the effect of oleanolic acid at the same concentration (10 *µ*M). However, lupeol and β-amyrin at a concentration of 10 *µ*M exhibited no significant effects on larval mass (Fig. 6B). These data suggest that *NaOSC1* and *NaOSC2* could impact *N. attenuata* defense against *M. sexta* through their direct products (e.g. β-amyrin and lupeol) or the oxidized derivatives of these products (e.g. betulinic acid and oleanolic acid), though the effect of betulinic acid appeared to be most dramatic.

### Metabolic consequences of *NaOSC1* and *NaOSC2* silencing in *N. attenuata*

Our analysis of product distribution across various tissues for *NaOSC1* and *NaOSC2* revealed that leaves are not the primary sites of accumulation for β-amyrin, lupeol, and the other unglycosylated products of *NaOSC1* (Fig. 4). To investigate whether the increase in larval mass is linked to the redirection of biosynthetic pathways toward sterol, as opposed to triterpene, synthesis following the silencing of *NaOSC1* or *NaOSC2*, we assessed the sterol and triterpene content in the leaves post-VIGS of *NaOSC1* or *NaOSC2*. Silencing of *NaOSC1* or *NaOSC2* did not significantly impact the levels of sterols (Supplemental Fig. S6). We observed a significant reduction (43% to 77%) in β-amyrin content in *NaOSC2*-silenced plants, with a slight decrease also observed in *NaOSC1* (*VIGS-NaOSC1-5*′*UTR_1*) plants. However, we did not find any other *NaOSC1* products in the leaves. Thus, we hypothesize that the defense mechanisms against *M. sexta* by *NaOSC1* and *NaOSC2* may predominantly stem from downstream, more intricate chemical components.

**Figure 7.**
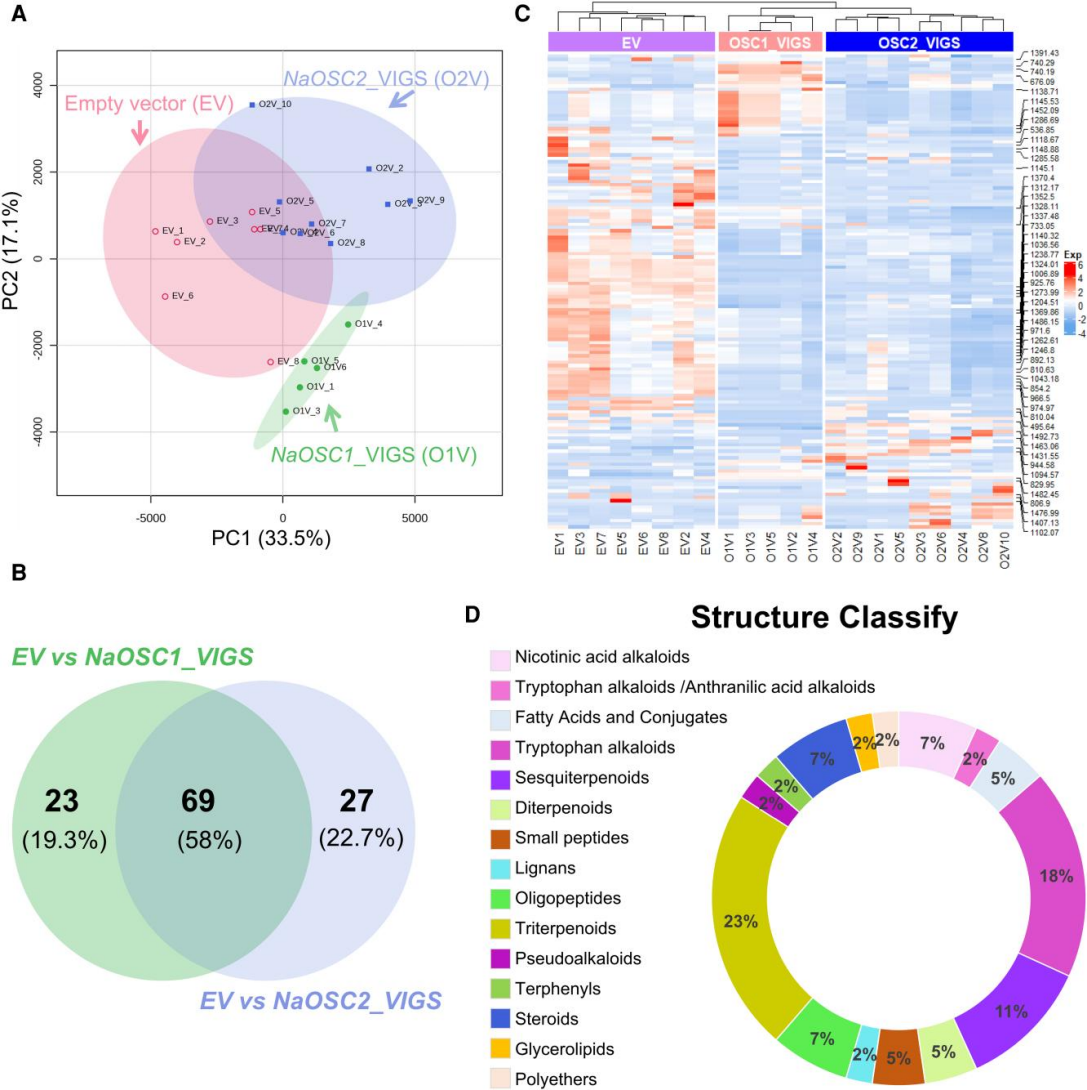
Untargeted metabolomic analysis on leaves of *NaOSC1*- and *NaOSC2*-VIGS plant. **A)** PCA score plot of metabolites. **B)** Venn diagram analysis of differential features. The left circle represents the number of features that significantly (Wilcoxon rank-sum test, *P* ≤ 0.05) changed only after *NaOSC1* silencing compared to EV, while the right circle represents the number of features that significantly (Wilcoxon rank-sum test, *P* ≤ 0.05) changed only after *NaOSC2* silencing compared to EV. The overlapping area between the left and right circles represents the number of features that exhibit differential changes when both *NaOSC1* and *NaOSC2* are silenced. **C)** The heatmap of features altered only after *NaOSC1* or *NaOSC2* silencing. **D)** The annotation of compound structural categories for differential features. Different colors represent the categories of annotated compounds, and the percentages on the ring indicate the proportion of that compound category among the total annotated features.

Untargeted LC-MS metabolite analyses on leaves of EV and *NaOSC1*-VIGS or *NaOSC2*-VIGS (EV, *NaOSC1-5*′*UTR_1*, and *NaOSC2-5*′*UTR_1*) plants were performed to explore the metabolic profile changes resulting from the silencing of NaOSCs. Principal component analysis (PCA) reveals differences among 3 groups of samples. Specifically, PC1 and PC2 contributed 33.5% and 17.1%, respectively, to the discrimination of the 3 sample groups (Fig. 7A). There were 92 features with significant differences between EV and *NaOSC1*-VIGS, while there were 96 features with significant differences between EV and *NaOSC2*-VIGS. Among them, 69 different features were shared between the 2 (Fig. 7B). Most of these features possess a larger molecular weight (>500) and exhibit a substantial downregulation in plants silenced for *NaOSC1* or *NaOSC2* (Fig. 7C). The features were annotated by the taxonomically informed scoring metabolite annotation method (Rutz et al. 2019). Among these differential features, 78% were successfully annotated, with 48% of the annotated differential features having distinct chemical classifications. Triterpenoids constituted the largest category at 23%, followed by tryptophan alkaloids at 18%, and sesquiterpenes, steroids, and diterpenes at 11%, 7%, and 5%, respectively (Fig. 7D).

### Feature-based molecular networking reveals potential triterpene glycoside clusters

**Figure 8.**
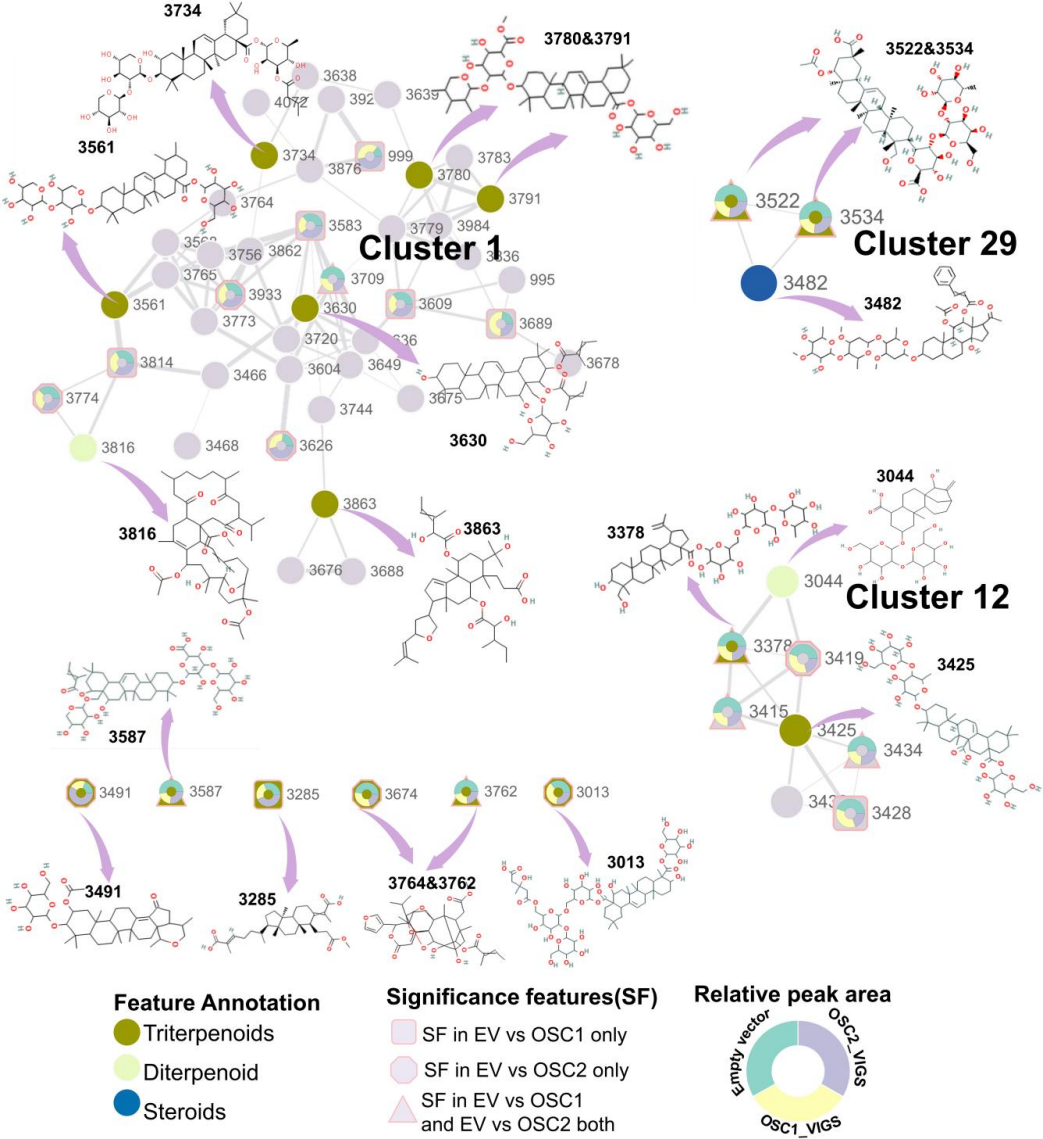
FBMN for triterpenoids. The MS2 files, processed using MetaboScape software, were imported into the GNPS platform. Pairwise spectral similarity between fragmentation spectra was computed using a modified cosine score, visualizing these relationships as a molecular network. Higher cosine scores indicate greater spectral similarity. During network construction, edges were filtered to retain those with cosine scores above 0.7 and more than 6 matching peaks. Each node represents a fragmentation spectrum. The thickness of the edges between nodes reflects the cosine scores, with thicker lines indicating higher cosine values and increased spectral similarity. SF, the significant feature obtained from Fig. 7 (Wilcoxon rank-sum test, *P* ≤ 0.05); EV, empty vector. Node text indicates feature IDs, while node color represents annotated compound types. Square nodes signify features with significant changes when *NaOSC1* is silenced compared to the control (EV). Octagonal nodes represent features with significant differences when *NaOSC2* is silenced. Triangular nodes depict features with significant changes compared to the control (EV), regardless of *NaOSC1* or *NaOSC2* silencing. Tricolor ring nodes show the relative abundance of features with significant differences in EV (green), NaOSC1_VIGS (yellow), and NaOSC2_VIGS (purple) plants.

GNPS, a web-based MS knowledge capture and analysis platform (https://gnps.ucsd.edu/), has been extensively employed in MS-based metabolomics to facilitate the annotation of molecular families based on their fragmentation spectra (MS2; Wang et al. 2016). GNPS incorporates an advanced computational tool, feature-based molecular networking (FBMN), which allows for quantitative analysis and isomer resolution of multiple LC-MS/MS files (Nothias et al. 2020). Utilizing FBMN, we identified a total of 30 clusters (Supplemental Fig. S7), and feature annotations of these clusters are shown in Supplemental Table S6. Three clusters (Cluster 1, Cluster 12, and Cluster 29) were characterized by the presence of distinct triterpene compounds (Fig. 8). Among these clusters, most of the annotated triterpene glycosides belong to the oleanane-type triterpenes. In Cluster 12, 1 compound was annotated as a lupane-type triterpene glycoside. Additionally, among the unclassified triterpene compounds, there are representatives of limonoids that belong to the class of tetranortriterpenoids (Fig. 8). We annotated the 144 selected features from the prior differential analysis in these molecular networks and observed that most features assigned to triterpenoid clusters exhibited a reduction following the silencing of *NaOSC1* or *NaOSC2* (Fig. 8). Additionally, we noted that after silencing of *NaOSC1*, a cluster annotated as sesquiterpenes exhibited an increase in features compared to the EV plants. In another cluster associated with alkaloids, these features decreased relative to the EV plants following *NaOSC2* silencing. Notably, in the cluster annotated as sterol glycosides, most features displayed a decrease after *NaOSC1* silencing, whereas they increased following *NaOSC2* silencing (Supplemental Fig. S8).

### Robust negative correlation between triterpene glycoside candidates and larval mass

**Figure 9.**
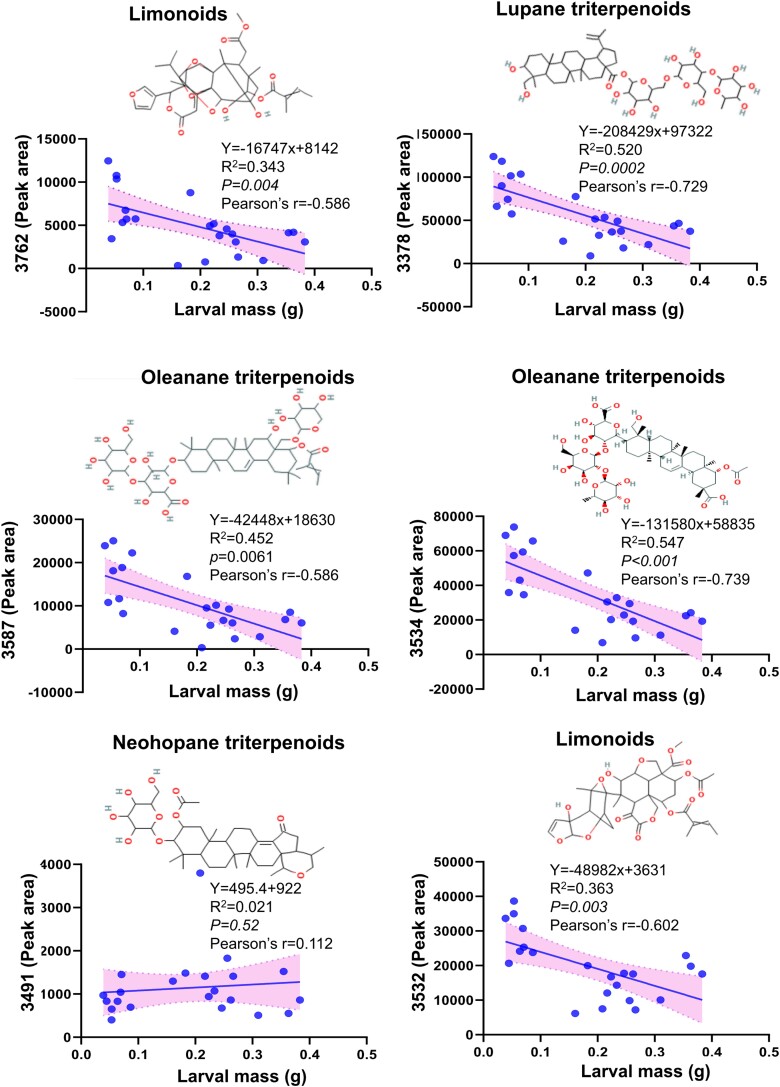
Correlation analysis between annotated triterpenoid features and larval mass. The fitting method employed is linear regression, and the correlation analysis method used is Pearson's correlation. *R*^2^ represents the degree to which the independent variable explains the variance in the dependent variable *y*, while Pearson’s *r* represents the Pearson correlation coefficient. The points represent specific observations in the data set. The lines reflect the connecting curve, and the colored zone reflects 95% confidence intervals.

The results described above clearly indicate that the silencing of *NaOSC1* or *NaOSC2* impacts accumulation of downstream triterpene glycosides. We conducted linear regression analyses between features annotated as triterpenes and larval mass to determine their relationship with larval growth. The calculation of correlation coefficients was conducted using Pearson's correlation analysis. We observed that the features annotated as triterpene compounds (3762, 3378, 3587, 3534, and 3532) all exhibited a significant negative correlation with larval mass (Fig. 9). Among them, feature 3534 displayed the strongest negative correlation with larval mass, with a Pearson correlation coefficient of −0.739. Following this, feature 3378, annotated as a lupane-type triterpene glycoside, showed a Pearson correlation coefficient of −0.729. Feature 3587, annotated as an oleanane-type triterpene glycoside, exhibited a negative correlation coefficient of −0.721. The two limonoid-type features displayed negative correlation coefficients of −0.586 (3762) and −0.602 (3532) with larval mass (Fig. 9). This suggests that the decrease in these features could be a key factor contributing to the observed increase in larval mass following the silencing of *NaOSC1* or *NaOSC2*.

## Discussion

Triterpenes are a large class of structurally diverse compounds. *N. attenuata*, an ecological model plant used for studying plant–herbivore interactions, has not been reported to contain triterpenes to date. Additionally, there is limited characterization of triterpene biosynthetic pathways in the *Nicotiana* genus, and the ecological functions of triterpenes in *Nicotiana* plants are generally unknown. The work presented here identified 4 complete OSC genes from the *N. attenuata* genome. Our characterization of these cyclases revealed the triterpene core scaffolds that are produced in *N. attenuata*. Subsequently, silencing these enzymes in *N. attenuata* has revealed their roles in defense against herbivores.

Phylogenetic analysis revealed that NaOSC1 and NaOSC2 cluster within a clade of β-amyrin synthases. NaOSC1 and NaOSC2 share the closest relationship with tomato (*S. lycopersicum*) enzymes, SlTTS1 and SlTTS2. SlTTS1 is characterized as a β-amyrin synthase, while SlTTS2 is a multifunctional enzyme primarily involved in δ-amyrin synthesis (Wang et al. 2011). In contrast, NaOSC4 and NaOSC5 fall into branches associated with lanosterol synthase and cycloartenol synthase, respectively. The product profiles of these NaOSCs were determined by transient heterologous expression of these genes in *N. benthamiana*, and notably, only NaOSC1 and NaOSC2 displayed activity. NaOSC1 synthesized 10 primary products, including dammarenediol II, lupeol, and 3-alpha,20-lupanediol (Fig. 3), with lupeol and 3-alpha,20-lupanediol coinciding as major products with those produced by AtLUP1, a multifunctional cyclase from *A. thaliana* (Segura et al. 2000). The minor products of NaOSC1, including δ-amyrin, β-amyrin, Ψ-taraxasterol, and taraxasterol, were also produced by SlTTS2 (Wang et al. 2011). *NaOSC2* produces β-amyrin as its sole product, consistent with the function of SlTTS1 in *S. lycopersicum* (Wang et al. 2011).

The immediate products of NaOSC1 and NaOSC2 primarily accumulate in young tissue organs, with the highest levels found in seedlings or young plant roots. Additionally, the products of *NaOSC*, β-amyrin and its derivatives, erythrodiol and oleanolic acid, accumulate in flowers at levels comparable to those in seedlings or young roots. RT-qPCR analysis confirmed that the expression of *NaOSC1* and *NaOSC2* is observed in roots, flowers, and trichomes.

Underivatized triterpenes are primarily found in the plant’s cuticular layer, where they serve as hydrophobic barriers or defense mechanisms (Jetter and Schaffer 2001; Guhling et al. 2006; Buschhaus and Jetter 2012). For example, elevated levels of β-amyrin in the Arabidopsis cuticle increase waterproofing (Buschhaus and Jetter 2012). Deficiencies in terpenes within the flowers can lead to the failure of rice pollen coat formation, resulting in humidity-sensitive male infertility (Xue et al. 2018). Moreover, simple terpenes may also regulate root development and promote the formation of root hairs (Kemen et al. 2014). Therefore, the spatiotemporal distribution specificity of simple terpenes in *N. attenuata* may be associated with varying water requirements, defense needs, or developmental demands in different stages and tissues.

Our study revealed an increased expression of *NaOSC1*, rather than *NaOSC2*, in response to *M. sexta* infestation in leaves. This led us to hypothesize that *NaOSC1* might play a more crucial role in defense against *M. sexta* attack. To investigate this further, we conducted VIGS to separately silence the expression of these 2 genes. Surprisingly, the results showed that silencing either *NaOSC1* or *NaOSC2* led to a significant susceptibility of *N. attenuata* to attack by *M. sexta* larvae (Fig. 6A). Additionally, in vitro toxicity assays demonstrated that the direct products as well as some of the oxidized derivatives of NaOSC1 and NaOSC2 could inhibit larval growth (Fig. 6B). However, despite the higher expression of *NaOSC1* and *NaOSC2* in *N. attenuata* trichomes, and the increase in NaOSC1 and NaOSC2 product levels in response to jasmonic acid signaling or herbivore infestation, we detected limited levels of their products in leaves or stems. This may be attributed to these triterpene unglycosylated products not being the primary forms accumulated in the plant or possibly being metabolized into more complex downstream structures.

To address this, we conducted a metabolomic analysis of the leaves. We observed that the metabolic profiles of leaves in which *NaOSC1* and *NaOSC2* were silenced were substantially distinct from those of EV plants. Furthermore, the features exhibiting metabolic changes were predominantly downregulated and possessed larger molecular weights. We utilized a metabolite annotation approach reported by Rutz et al. (2019), which integrates various metabolite annotation tools, including ISDB-DNP (Allard et al. 2016), MS-Finder (Vaniya et al. 2017), and Sirius (Dührkop et al. 2019), to annotate the features we detected. Subsequently, these features were categorized into distinct molecular network clusters using FBMN. Among the features that exhibited differences compared to the EV control, 78% were successfully annotated. Notably, within this group, 23% were identified as belonging to the triterpene class. Additionally, there were some features that, while not directly annotated, clustered together with the annotated triterpene features within the same network cluster, indicating a potential shared structural profile among these features.

Among the triterpenoid features that we annotated using this method, we observed oleanane-type saponins, lupinane saponins, and limonoids. Notably, all these features exhibited a significant negative correlation with larval mass. Pentacyclic triterpenoid glycosides or limonoids have been reported to possess antifeedant and insecticidal properties (Alford et al. 1987; Gao et al. 2011; Li et al. 2013; Wang et al. 2020). For instance, studies have demonstrated the efficacy of limonin against the Colorado potato beetle and *Pieris rapae* (Alford et al. 1987; Wang et al. 2020). Oleanane or lupinane saponins exhibit antifeedant effects on diamondback moth larvae, flea beetles, and nematodes (Taylor et al. 2004; Gao et al. 2011; Li et al. 2013). This suggests that the observed larval performance resulting from the silencing of *NaOSC1* or *NaOSC2* may be associated with changes in downstream complex triterpenoid compounds.

In summary, we discovered the enzyme that catalyzed the first committed step in triterpenoid biosynthesis in *N. attenuata* plants. Through VIGS and in vitro bioassays, we have confirmed the pivotal roles that these enzymes, NaOSC1 and NaOSC2, play in the defense mechanisms of *N. attenuata* against herbivores. Furthermore, by constructing molecular networks and profiling metabolite changes induced by *NaOSC1* or *NaOSC2* silencing, we have identified potential triterpene glycoside molecular clusters, suggesting the presence of additional unknown triterpenoid compounds in *N. attenuata*. This study provides the characterization of triterpene metabolism in the important model plant *N. attenuata*.

## Materials and methods

### Chemicals

α-Amyrin, β-amyrin, lupeol, cycloartenol, betulinic acid, oleanolic acid, dichloroisocyanuric acid, salicylic acid, methyl jasmonate, and abscisic acid were purchased from Sigma-Aldrich (St Louis, MO, USA). Dammarenediol II was purchased from ChemFaces (Wuhan, China). Gibberellin A_3_ was purchased from Carl Roth (Karlsruhe, Germany).

### Plant materials and growth conditions

*N. attenuata* Torr. Ex Watts seeds of the 31st-generation inbred line were used as the wild-type (WT) genotype in all experiments. Seed germination and plant growth were performed as described (Krügel et al. 2002), with a day/night cycle of 16 h (26 to 28 °C) and 8 h (22 to 24 °C) in a glasshouse at the Max Planck Institute for Chemical Ecology, Jena, Germany.

### Bioinformatic analysis

Sequences of proteins encoded by the *N. attenuata* genome were downloaded from NCBI (https://www.ncbi.nlm.nih.gov/genome/). A public hidden Markov model (PF13243, http://pfam.xfam.org/) was used to predict the OSCs in *N. attenuata* (Fan et al. 2022). All the hits with amino acid sequence lengths greater than 600 were selected for functional identification. Multisequence alignment and visualization were performed by ClustalW (https://www.genome.jp/tools-bin/clustalw) and ENDscript/ESPript (https://espript.ibcp.fr/ESPript/cgi-bin/ESPript.cgi). The neighbor-joining tree was constructed and tested by bootstrapping (1,000 times) by MEGA11. Accession numbers of the related triterpene synthase sequences are listed in Supplemental Table S2. The embellishment of the phylogenetic tree was carried out using the tvBOT tool (Xie et al. 2023).

### Elicitation by phytohormones

*N. attenuata* Torr. Ex Watts seeds of the 31st-generation inbred line were surface sterilized with 1 M dichloroisocyanuric acid for 5 min and then washed 3 times with sterile water. The sterilized seeds were incubated for 1 h with the germination cues, which contained 0.1 *μ*M gibberellin A_3_ (Carl Roth) and 50-fold diluted liquid smoke (House of Herbs, Passaic, NJ). Incubated seeds were washed 3 times with sterile water and sown on agar-solidified Gamborg’s B5 medium (pH 6.8) with a nylon mesh (mesh size 20 *µ*m, Prosep) and incubated vertically in a plant culture chamber at 26 °C under long-day conditions (16-h light/8-h dark) for 14 d. The 14-d-old seedlings were transferred to Gamborg’s B5 solid medium with methyl jasmonate (100 *μ*M), salicylic acid (100 *μ*M), gibberellin A_3_ (100 *μ*M), or abscisic acid (100 *μ*M). Medium without hormones was used as a negative control. Seedlings were harvested at 0, 3, 6, and 9 h after transfer for RNA extraction. For *M. sexta* larvae elicitation, the leaves of 4-wk-old plants were infested with newly hatched larvae, and the infested leaves were harvested at time points of 0, 0.5, 1, 3, 6, 9, and 24 h. The treatment method for methyl jasmonate in 4- and 8-wk-old plants involved applying 250 *μ*g of methyl jasmonate in lanolin to the adaxial surface of 3 leaves, 3 lateral branches, and stems (approximately 3 cm above ground) for a duration of 72 h. Plants treated with an equivalent amount of lanolin without methyl jasmonate served as the control. The roots, stem barks, flower buds, blooming flowers, and leaves of each plant were collected for metabolite analysis.

### Functional characterization of OSCs in *N. benthamiana*

Full-length cDNAs of selected genes (*NaOSCs* from *N. attenuata*) and *AtLup1* (from Arabidopsis [*A. thaliana*]) were cloned (digested with BsaI) into a modified 3Ω1 expression vector (Cárdenas et al. 2019) using the ClonExpress II One Step Cloning Kit (Vazyme) and transformed into *A. tumefaciens* strain GV3101. The primers used for amplification are listed in Supplemental Table S3. *Agrobacterium* strains containing the gene constructs were grown for 24 h at 28 °C in 10 mL of LB medium with antibiotics (100 *μ*g mL^−1^ rifampicin and 250 *μ*g mL^−1^ spectinomycin) and then centrifuged at 2,000 × *g* for 20 min, and the supernatant was removed. The cell pellet was resuspended in 5 mL of infiltration buffer (50 mM MES, 2 mM Na_3_PO_4_, 10 mM MgCl_2_, and 100 *μ*M acetosyringone) and diluted to an optical density OD_600_ of 0.6. Following a 2-h incubation at room temperature, infiltration was carried out on the lower surface of 4- to 5-wk-old *N. benthamiana* leaves using a needleless 1-mL syringe. Five days after infiltration, the leaves of 3 different plants were collected for triterpenoid analysis.

### VIGS

We PCR amplified 250- to 300-bp segments from the 5′UTR or 3′UTR regions of NaOSCs using Q5 High-Fidelity DNA Polymerase (New England Biolabs) and the primers specified in Supplemental Table S4. Subsequently, these fragments were inserted into *pTV00* and introduced into *A. tumefaciens* GV310. We infiltrated the leaves of young *N. attenuata* plants with *pBINTRA* and *pTV-NaOSCs*, following a published protocol optimized for VIGS in *N. attenuata* (Saedler and Baldwin 2004). Plants coinfiltrated with *pBINTRA* and *pTV00* were used as negative controls, and plants coinfiltrated with *pBINTRA* and *pTV-PDS* were used as positive controls.

### Total RNA extraction and transcript abundance analysis

Total RNA was isolated from plant tissues of *N. attenuata* using the plant RNA purification kit (Macherey-Nagel) according to the manufacturer's instructions. PrimeScript RT Master Mix (Takara Bio Inc., Japan) was used to synthesize cDNA from total RNA. RT-qPCR was performed on a Stratagene Mx3005P qPCR machine using a Takyon No ROX SYBR 2X MasterMix Blue dTTP (Eurogentec, Seraing, Belgium). The housekeeping gene IF-5α from *N. attenuata* was used as an internal reference. The primers used for RT-qPCR are listed in Supplemental Table S5.

### Insect performance assay

*M. sexta* performance assays were conducted on *NaOSCs-VIGS* plants, as described earlier (Pradhan et al. 2017; Heiling et al. 2021). Individual replicates (*n* = 15) of each *NaOSC-VIGS* and empty vector plant were used. Plants were grown in a climate chamber (55% to 65% relative humidity, 24 to 26 °C day, and 23 to 25 °C night, under 16 h of light), as described above in a randomized design. Neonates were positioned on the lower surface of the second fully developed leaf of rosette-stage plants, and larvae were allowed to feed for 13 d before recording caterpillar mass.

### Larval bioassay with artificial diets

The artificial diet was prepared as previously reported (Machado et al. 2015) with slight modifications. Briefly, 6.67 g wheat germ, 1.33 g yeast extract, 2.67 g sucrose, 1 g Wesson salt mixture, 0.41 g ascorbic acid, 0.29 g cholesterol, 0.17 g sorbic acid, 83 mg methylparaben, 0.33 mL raw linseed oil, 8.3 mg streptomycin, 8.3 mg kanamycin, 1 mL formalin, and 1.2 mL vitamin mixture (100 mg nicotinic acid, 500 mg riboflavin, 233.5 mg thiamin, 233.5 mg pyridoxine, 233.5 mg folic acid, and 20 mg L^−1^ biotin in water) were added to 4 × 9 cm plastic boxes. Agar (34 g) was dissolved in 1,000 mL of water and sterilized at 121 °C for 20 min. Then, 65 mL of cooled agar medium (50 °C) was added to the box and stirred. The β-amyrin, lupeol, oleanolic acid, and betulinic acid were dissolved in *N*,*N*-dimethylformamide (DMF) and added to the diet mixture. A diet mixture containing an equal amount of triterpene-free DMF was used as a negative control. After melting, the diet was aliquoted into small plastic boxes and kept at 4 °C until use. Diets were freshly prepared and replaced every other day. Larvae (5 larvae per plate; 5 plates per diet type) were fed ad libitum in a climate chamber (45% to 55% relative humidity, 24 to 26 °C during days, and 23 to 25 °C during nights under 16 h of light). Larval mass was determined as described earlier at 7, 9, and 11 d after the beginning of the experiment (*n* = 25).

### Metabolite extraction and GC-MS analysis

The process of extraction of triterpenes was based on previously published methods (Field and Osbourn 2008) with small modifications. Approximately 1 g of the fresh, unground tissue (leaf disks with a diameter of 2 cm, 1 cm of stem bark, 1 cm of root, and intact flower buds and blooming flower) was washed in 10 mL hexane, which contained 200 ng α-amyrin as internal standard, for 1 min to extract the triterpenes. The extracts were vacuum dried and saponified in 300 *μ*L 20% KOH (w/v) in 50% EtOH (v/v) with 0.5 mg mL^−1^ butylated hydroxytoluene (Sigma-Aldrich) for 2 h at 65 °C and extracted 3 times with 300 *μ*L hexane. Following hexane extraction, 100 *μ*L of 10 M HCl was added to the aqueous solution to lower the pH below 2.0, and another round of hexane extractions was performed to obtain an acid extraction fraction. The alkaline and acid hexane extracts were concentrated and derivatized with a mixture of *N*-methyl-*N*-trimethylsilyl-trifluoroacetamide (MSTFA) and DMF before GC-MS analysis.

The extraction of triterpenes or sterols from *N. benthamiana* leaves heterologously expressing OSCs was performed following the same procedure as previously described. However, in this case, the saponification buffer was directly added to 100 mg of frozen ground tissue powder, and 10 *μ*L of α-amyrin with a concentration of 10 ng *μ*L^−1^ was added to the samples as an internal standard. The mixture was then incubated at 65 °C for 2 h. GC-MS analyses were conducted on a CP-3800 GC Varian Saturn 4000 ion trap mass spectrometer (Varian) connected to a nonpolar SLB-5ms capillary column (30-m × 0.25-mm i.d., 0.25-*μ*m film thickness; Sigma). Samples (1 *µ*L) were injected by a CP-8400 autoinjector (Varian) in splitless mode. The column temperature was initially set at 180 °C and held for 1 min and then ramped with 6 °C·min^−1^ to 230 °C and held for 2 min; the temperature was subsequently increased to 260 °C with 3 °C min^−1^ and held for 15 min and finally with 8 °C·min^−1^ to 320 °C and held for 10 min. Helium carrier gas was used, and the column flow was set to 1 mL·min^−1^. Eluted compounds from the gas chromatograph column were transferred to a Varian Saturn 4000 ion trap mass spectrometer for analysis, and the mass spectral data were acquired for the duration of the GC-MS program from *m*/*z* 50 to 600.

### LC-MS detection and metabolite analysis

Extraction and chromatographic analysis procedures for specialized metabolites were based on previously published methods (Schäfer et al. 2016). Briefly, 100 mg of frozen ground leaf tissue was extracted with 1 mL precooled (−20 °C) 80% methanol (v/v) containing internal standards (40 *μ*g·mL^−1^ digitoxin). For the chromatographic separation of metabolites, 1 *μ*L of each sample was subjected to separation using an AcclaimC18 column (150 × 2.1 mm, 2.2 *μ*m particle size, Thermo Fisher Scientific) coupled with a UPLC SecurityGuard ULTRA cartridge (Phenomenex, catalog #: AJO-8782). The mobile phase consisted of eluent A (deionized water containing 0.05% formic acid [v/v] and 0.1% acetonitrile [v/v]) and eluent B (acetonitrile containing 0.05% formic acid [v/v]) at a flow rate of 0.400 mL·min^−1^. The gradient elution protocol was as follows: 0 to 0.5 min, isocratic elution with 90% A; 0.5 to 23.5 min, a linear gradient from 90% to 10% A; and 23.5 to 25 min, isocratic elution with 10% A. MS detection was conducted using an impact II Q-TOF-MS system (Bruker Daltonics, Bremen, Germany) with an electrospray ionization (ESI) source operating in positive ion mode. Nitrogen served as the nebulizer, drying and collision gas, with a capillary voltage of 4,500 V and an end plate offset of 500 V. The nebulizer pressure was set to 1.4 bar, dry gas flow rate was 10 L·min^−1^, and the temperature was maintained at 200 °C. Full scan data were recorded from *m*/*z* 50 to 1,500, and data-dependent MS/MS acquisitions were carried out on the 3 ions with the highest intensity identified in the full-scan MS using the autoMS/MS function (Bruker). The MS/MS isolation window width was set dependent on the parent ion *m*/*z* from 8 Da (*m*/*z* 500) to 15 Da (*m*/*z* 2,000), and the collision energy was set to 30 eV.

### Metabolomic and FBMN analysis

For the metabolomic analysis, the raw data files were processed using MetaboScape 5.0 (Bruker) for peak alignment, deconvolution, and extraction of MS2 features. Subsequently, only features associated with MS2 scans were retained. A quantitative table and spectral file (.mgf) were exported for the construction of a molecular network and statistical analysis. To ensure the retention of retention time, exact mass information, and separation of isomers, a feature-based molecular network was generated on the GNPS web-based MS knowledge capture and analysis platform (https://gnps.ucsd.edu/; Wang et al. 2016) using the output files generated by MetaboScape 5.0. The spectra within the network were searched against GNPS’ spectral libraries. To be included, matching between network spectra and library spectra necessitated a score exceeding 0.7, along with a minimum of 6 matched peaks. The results were visualized using Cytoscape (version 3.10.1). For the annotation of features, a method reported by Rutz et al. (2019) was employed. In brief, the spectral file and attributes metadata obtained after the molecular network analysis were annotated using the ISDB-DNP with the following parameters: parent mass tolerance of 0.005 Da, minimum cosine score of 0.2, and a maximum of 50 returned candidates. Subsequently, an R script provided by https://github.com/oolonek/taxo_scorer was utilized for taxonomically informed scoring on GNPS outputs, resulting in an attribute table.

The quantification table after the FBMN step was also submitted to MetaboAnalyst 5.0 for PCA and the identification of significant features. Prior to analysis, the data underwent normalization with a log transformation (Base 10) and Pareto scaling (mean centered and divided by the square root of the Sd). Significant MS features were determined using 2-sample *t* tests and Wilcoxon rank-sum tests. Heatmap analyses were conducted using R packages complexheatmap, corrplot, and psych.

### Statistical analysis

Statistical analysis and figure generation were carried out using GraphPad Prism 9.2 (GraphPad Software, La Jolla, CA, USA), IBM SPSS Statistics 23 (IBM Inc., Chicago, IL, USA), and OriginPro 2022 (OriginLab Corporation, Northampton, MA, USA). Statistical differences among groups were determined using ANOVA followed by Tukey’s honestly significant difference (HSD) post hoc test. The significance of differences between 2 sample groups was assessed using Student's *t* test. A significance level of *P* < 0.05 was considered statistically significant for all comparisons.

### Accession numbers

Sequence data from this article can be found in the GenBank/EMBL data libraries under accession numbers *NaOSC1* (LOC109226501), *NaOSC2* (LOC109226503), *NaOSC3* (LOC109239410), *NaOSC4* (LOC109242372), *NaOSC5* (LOC109241809), and *NaOSC6* (LOC109230529).

## Supplementary Material

kiad643_Supplementary_Data

## Data Availability

All data are incorporated into this article and its online supplementary material.

## Supplemental data

The following materials are available in the online version of this article.

**Supplemental Figure S1.** A motif comparison of OSCs in different species.

**Supplemental Figure S2.** EI mass spectra of the products generated by *NaOSC1* or *NaOSC2*.

**Supplemental Figure S3.** Heterologous expression of *NaOSC4* and *NaOSC5* in *N. benthamiana*.

**Supplemental Figure S4.** Extracted ion chromatogram (*m*/*z* 189, 203, 218, 199, and 131) of triterpenes in different tissues.

**Supplemental Figure S5.** Silencing efficiency of *NaOSC1* and *NaOSC2* in NaOSC-VIGS plants.

**Supplemental Figure S6.** Triterpene and sterol content in NaOSC-VIGS plants.

**Supplemental Figure S7.** An overview of FBMN.

**Supplemental Figure S8.** FBMN for carbazole alkaloids, sesquiterpenes, and steroids.

**Supplemental Table S1.** Biochemical information of OSC candidate genes.

**Supplemental Table S2.** OSC sequences used for phylogenetic analysis.

**Supplemental Table S3.** Primer sequences used for the design of constructs for transient expression.

**Supplemental Table S4.** Primer sequences used for VIGS vectors.

**Supplemental Table S5.** Primer sequences used for RT-qPCR.

**Supplemental Table S6.** Metabolite annotation table.

## Dive Curated Terms

The following phenotypic, genotypic, and functional terms are of significance to the work described in this paper:

betulinic acid CHEBI: CHEBI:3087

triterpenes CHEBI: CHEBI:35191
