# Supplementary material for: OXIDOSQUALENE CYCLASE 1 and 2 influence triterpene biosynthesis and defense in Nicotiana attenuata
Source: Plant Physiol. 2023 Dec 15;194(4):2580–99. doi: 10.1093/plphys/kiad643 (PMC10980520; doi:10.1093/plphys/kiad643)
Supplement: kiad643_Supplementary_Data [file kiad643_supplementary_data.zip › Supplemental data.pdf]

Supplemental Information

Article title: **OXIDOSQUALENE CYCLASE 1 and 2 influence triterpene biosynthesis and defense in *Nicotiana attenuata***

Authors: Caiqiong Yang, Rayko Halitschke, Sarah E. O'Connor

The following supplemental tables and figures are available for this article:

Supplemental Table S1 Biochemical information of OSC candidate genes

Lable	Gene name	CDS (bp)	Number of Exons	length (aa)	PI	MW(KDa)
NaOSC1	LOC109226501	2273	18	757	5.68	86.54
NaOSC2	LOC109226503	2285	21	761	5.71	87.48
NaOSC3	LOC109239410	2096	18	698	5.89	79.66
NaOSC4	LOC109242372	2306	19	768	6.38	88.13
NaOSC5	LOC109241809	2447	22	815	6.29	92.55
NaOSC6	LOC109230529	419	4	139		15.98

Supplemental Table S2 OSC sequences used for phylogenetic analysis

OSCs	GenBank ID	Plant	Product	*Ref.*
LsOSC2	MG708183	*Lagerstroemia speciosa*	α-Amyrin	(Sandeep *et al.*, 2019)
OeOEA	AB291240	*Olea europaea*	α-Amyrin	(Saimaru *et al.*, 2007)
SlTTS1	HQ266579	*Solanum lycopersicum*	*β*-Amyrin	(Wang *et al.*, 2010a)
MtAMYI	AJ430607	*Medicago truncatula*	*β*-Amyrin	(Iturbe-Ormaetxe *et al.*, 2003)
AsbAS1	AJ311789	*Avena strigosa*	*β*-Amyrin	(Haralampidis *et al.*, 2001)
VhBS	DQ915167	*Vaccaria hispanica*	*β*-Amyrin	(Meesapyodsuk *et al.*, 2007)
PtbAS	EF107623	*Polygala tenuifolia*	*β*-Amyrin	(Jin *et al.*, 2014)
PsPSY	AB034802	*Pisum sativum*	*β*-Amyrin	(Morita *et al.*, 1997)
PgPNY2	AB014057	*Panax ginseng*	*β*-Amyrin	(Kushiro *et al.*, 1998)
PgPNY1	AB009030	*Panax ginseng*	*β*-Amyrin	(Kushiro *et al.*, 1998)
LjAMY1	AB181244	*Lotus japonicus*	*β*-Amyrin	(Sawai *et al.*, 2006b)
GgbAS1	AB037203	*Glycyrrhiza glabra*	*β*-Amyrin	(Hayashi *et al.*, 2001)
EtAS	AB206469	*Euphorbia tirucalli*	*β*-Amyrin	(Kajikawa *et al.*, 2005)
BpBPY	AB055512	*Betula platyphylla*	*β*-Amyrin	(Zhang *et al.*, 2003)
BgbAS	AB289585	*Bruguiera gymnorrhiza*	*β*-Amyrin	(Basyuni *et al.*, 2006)
AtLUP4	At1g78950	*Arabidopsis thaliana*	*β*-Amyrin	(Shibuya *et al.*, 2009)
AsOXA1	AY836006	*Aster sedifolius*	*β*-Amyrin	(Cammareri *et al.*, 2008)
AabAS	EU330197	*Artemisia annua*	*β*-Amyrin	(Kirby *et al.*, 2008)
RcCAS	DQ268870	*Ricinus communis*	Cycloartenol	(Guhling *et al.*, 2006a)
MiOSC1	APG38074.1	*Maytenus ilicifolia*	Cycloartenol	(Souza-Moreira *et al.*, 2016)
GgCAS1	AB025968	*Glycyrrhiza glabra*	Cycloartenol	(Hayashi *et al.*, 2004)
CpCPX	AB116237	*Cucurbita pepo*	Cycloartenol	(Shibuya *et al.*, 2004)
AtCAS1	At2g07050	*Arabidopsis thaliana*	Cycloartenol	(Corey *et al.*, 1993)
RsCAS	AB292608	*Rhizophora stylosa*	Cycloartenol	(Basyuni *et al.*, 2007)
PgPNZ1	AB009031	*Panax ginseng*	Lanosterol	(Suzuki *et al.*, 2006)
LjLAS	AB244671	*Lotus japonicus*	Lanosterol	(Sawai *et al.*, 2006a)
AtLSS1	At3g45130	*Arabidopsis thaliana*	Lanosterol	(Kolesnikova *et al.*, 2006)
RcLUS	DQ268869	*Ricinus communis*	Lupeol	(Guhling *et al.*, 2006b)
LjOSC3	AB181245	*Lotus japonicus*	Lupeol	(Sawai *et al.*, 2006b)
KdLUS	HM623871	*Kalanchoe daigremontiana*	Lupeol	(Wang *et al.*, 2010b)
GgLUS1	AB116228	*Glycyrrhiza glabra*	Lupeol	(Hayashi *et al.*, 2004)
ToTRW	AB025345	*Taraxacum officinale*	Lupeol	(Shibuya *et al.*, 1999)
OeOEW	AB025343	*Olea europaea*	Lupeol	(Shibuya *et al.*, 1999)
BpBPW	AB055511	*Betula platyphylla*	Lupeol	(Zhang *et al.*, 2003)
BgLUS	AB289586	*Bruguiera gymnorrhiza*	Lupeol	(Basyuni *et al.*, 2007)
AtMRN1	At5g42600	*Arabidopsis thaliana*	Marneral	(Xiong *et al.*, 2006)
LjAMY2	AF478455	*Lotus japonicus*	Mixed products	(Iturbe-Ormaetxe *et al.*, 2003)
CsOSC2	AB058508	*Costus speciosus*	Mixed products	(Kawano *et al.*, 2002)
AtPEN6	At1g78500	*Arabidopsis thaliana*	Mixed products	(Ebizuka *et al.*, 2003)
SlTTS2	HQ266580	*Solanum lycopersicum*	Mixed products	(Wang *et al.*, 2011)

Supplemental Table S3 Primer sequences used for the design of constructs for transient expression

Name	Sequence 5' to 3'
OSC1-3Ω1-F	TTTATGAATTTTGCAGCTCGATGTGGAAATTGAAGATTGCACAA
OSC1-3Ω1-R	GACAACCACAACAAGCACCGTTAGTTGTTTTGTAAAACATTTTTG
OSC2-3Ω1-F	TTTATGAATTTTGCAGCTCGATGTGGAAGTTGAAGATTGCAGAA
OSC2-3Ω1-R	GACAACCACAACAAGCACCGTTAGTTGTTTTGTAATGGTGATAGG
OSC4-3Ω1-F	TTTATGAATTTTGCAGCTCGATGTGGAAACTTAAACTCTCAGAG
OSC4-3Ω1-R	GACAACCACAACAAGCACCGTTAATTCGAAGTGTTGGCTATTTC
OSC5-3Ω1-F	TTTATGAATTTTGCAGCTCGATGTGGAAGTTGAAGATAGCAGA
OSC5-3Ω1-R	GACAACCACAACAAGCACCGCTAGCCAAAAAGATTGGCCATATT
lup1-3Ω1-F2	TTTATGAATTTTGCAGCTCGATGTGGAAGTTGAAGATAGGAAAGGGAAATG
lup1-3Ω1-R2	GACAACCACAACAAGCACCGTTAATTAACGATAAACACAACTTTTCGGTA

Supplemental Table S4 Primer sequences used for VIGS vectors

Name	Sequence 5' to 3'
OSC1-SalI-5'UTR_1-F	GCGGCGGTCGACATCTCACTGGATTGTTGTTGTTG
OSC1-BamHI-5'UTR_1-R	GCGGCGGGATCCTCTTCTTCTAACCTTCTAGCTTACTG
OSC2-SalI-5'UTR_1-F	GCGGCGGTCGACCAACCTACCAAACAAAGGTATCC
OSC2-BamHI-5'UTR_1-R	GCGGCGGGATCCTTCTTAACACTTTCTTCTAGCTT
OSC1-SalI-5'UTR2-F	GCGGCGGTCGACATACCATGCATTTTTTTTATCACTTTAT
OSC1-BamHI-5'UTR2-R	GCGGCGGGATCCAACATAAAACTATATTATGTATATGCAG
OSC2-SalI-5'UTR2-F	GCGGCGGTCGACATTTGATGAATTTTAAACAACAAATAC
OSC2-BamHI-5'UTR2-R	GCGGCGGGATCCTCTTAACACTTTCTTCTAGCTTTTACT

Supplemental Table S5 Primer sequences used for RT-qPCR

Name	Sequence 5' to 3'
OSC1-F	CTAGCTCGTCAATCCAAGCA
OSC1-R	AAGCACACACCCCAGTTACC
OSC2-F	TGGCTGAATACCGCAAAAATGTCC
OSC2-R	TTGGGGGTAAACAGTAAGGACCAA
IF5a-F	GTCGGACGAAGAACACCATT
IF5a-R	CACATCACAGTTGTGGGAGG

GenBank ID	product	MWCYCR motif	FIKKSQ motif	QxxxxxW motif	QxxxxxW motif	QxxxxxW motif	QxxxxxW motif	DCTAE motif
XP_019246818.1_NaOSC1	unknown	MWCY**T**R	FI**NK**SQ	QNEDGGW	QASDGHW	QMPDGSW	QRS-DGGW	DCTAE
XP_019246822.1_NaOSC2	unknown	MWCYCR	FIKESQ	QNEDGGW	QASDGHW	QMPDGSW	QRS-DGGW	DCTAE
ADU52574.1_SlTTS1	β-amyrin	MWCYCR	FIKQSQ	QNEDGGW	QATDGHW	QMPDGSW	QRS-DGGW	DCTAE
XP_019261522.1_NaOSC3	unknown	MWCYCR	FIKKSQ	QNEDGGW	QASDGHW	QRPDGSW	QRS-DGGW	DCTAE
ADU52575.1_SlTTS2	multiple products	MWCYCR	FIKQSQ	QNEDGGW	QATDGHW	QEPDGSW	QRS-DGGW	DCTAE
NP_001323419.1_AtLUP4	β-amyrin	MWCYCR	FIKASQ	QNEDGGW	QADDGHW	QMPGGSW	QLK-DGGW	DCTAE
ACA13386.1_AabAS	β-amyrin	MWCYCR	FIKASQ	QNEDGGW	QADDGHW	QMEDGSW	QLE-DGGW	DCTAE
BAA33722.1_PgPNY2	β-amyrin	MWCYCR	FIKKSQ	QNDDGGW	QADDGHW	QMPDGSW	QRS-DGGW	DCTAE
BAA33461.1_PgPNY1	β-amyrin	MWCYCR	FIKKSQ	QNEDGGW	QASDGHW	QMPDGSW	QMD-DGGW	DCTAE
CAD23247.1_MtAMYI	β-amyrin	MWCYCR	FIKKSQ	QNEDGGW	QTSDGHW	QTADGSW	QRE-DGGW	DCTAE
BAA97558.1_PsPSY	β-amyrin	MWCYCR	FIKKSQ	QNEDGGW	QTSDGHW	QTEDGSW	QRE-DGGW	DCTAE
BAE53429.1_LjAMY1	β-amyrin	MWCYCR	FIKKSQ	QNEDGGW	QTSDGHW	QTADGSW	QRG-DGGW	DCTAE
AAO33580.1_LjAMY2	multiple products	MWCYCR	FIKNSQ	QNEDGGW	QTSDGHW	QTADGSW	QSK-DGGW	DCTAE
BAA89815.1_GgbAS1	β-amyrin	MWCYCR	FIKKSQ	QNEDGGW	QTSDGHW	QTADGSW	QRE-DGGW	DCTAE
ABL07607.1_PtbAS	β-amyrin	MWCYCR	FIKKSQ	QNEDGGW	QSSHGHW	QTADGSW	QKE-DGGW	DCTAE
ABK76265.1_VhBS	β-amyrin	MWCYCR	FIKESQ	QNEDGGW	QAEDGHW	QYPNGSW	QKE-DGGW	DCTAE
BAE43642.1_EtAS	β-amyrin	MWCYCR	FIKKSQ	QNEDGGW	QASDGHW	QTADGGW	QKQ-DGGW	DCTAE
CAC84558.1_AsbAS1	β-amyrin	FWCFTR	FMKNSQ	QNEDGGW	QADDGHW	QNKDGSW	QQT-TGGW	DCTAE
BAF80443.1_BgbAS	β-amyrin	MWCYCR	FIKKSQ	QNEDGGW	QASDGHW	QTSDGGW	QRD-NGGW	DCTAE
BAB83088.1_BpBPY	β-amyrin	MWCYCR	FIKKSQ	QNEDGGW	QASDGHW	QMPDGSW	QRD-NGGW	DCTAE
ADK35126.1_KdLUS	lupeol	MWCYCR	FIKKSQ	QNEDGGW	QASDGHW	QMPDGSW	QLE-DGGW	DCTAE
ABB76766.1_RcLUS	lupeol	MFCYCR	FTKNSQ	QNEDGGW	QASDGHW	QEPDGSW	QRD-DGGW	DCTAE
BAF80444.1_BgLUS	lupeol	MLCYCR	FIKNSQ	PNEDGGW	QASDGHW	QQPDGSW	QRD-DGGW	DCTAE
NP_199074.1_AtMRN1	marneral	LWIYIR	FLKQSQ	QNEDGGW	QANDGHW	QMPDGSW	QNA-EGGW	DGTAE
BAF63702.1_OeOEA	α-amyrin	MWCYCR	YVKESQ	QNDDGGW	QASDGHW	QWPDGSW	QNE-EGGW	DCTAE
BAE53430.1_LjOSC3	lupeol	MLCYCR	FVKASQ	QNEDGGW	QAHDGHW	QNPDGSW	QLP-NGGW	DCTAE
BAD08587.1_GgLUS1	lupeol	MLCYCR	FVKASQ	QNEDGGW	QAHDGHW	QNPDGSW	QLP-NAGW	DCTAE
BAB83087.1_BpBPW	lupeol	MLCYCR	FVKASQ	QNEDGGW	QAHDGHW	QEPDGSW	QLP-NGGW	DCTAE
BAA86932.1_ToTRW	lupeol	MLCYCR	FVKASQ	QNEDGGW	QAHDGHW	QESDGSW	QLP-DGGW	DCTAE
BAA86930.1_OeOEW	lupeol	MLCYCR	FVKASQ	QNEDGGW	QAHDGHW	QNPDGSW	QLP-DGGW	DCTAE
XP_019264148.1_NaOSC5	unknown	MWCHCR	FIKNTQ	QNSDGGW	QTLDGHW	QEADGSW	QIS-SGGW	DCTAE
ABB76767.1_RcCAS	cycloartenol	MWCHCR	FIKKMQ	QNRDGGW	QADDGHW	QISDGSW	QCP-SGGW	DCTAE
BAA76902.1_GgCAS1	cycloartenol	MWCHCR	YIKNSQ	QNKDGGW	QSHDGHW	QASDGSW	QLP-SGGW	DCTAE
NP_001325220.1_AtCAS1	cycloartenol	MWCHCR	FVKNSQ	QNEDGGW	QAHDGHW	QAADGSW	QQP-SGGW	DCTAE
BAD34644.1_CpCPX	cycloartenol	MWCHCR	YIKDSQ	QNKDGGW	QADDGHW	QATDGSW	ELA-AGGW	DCTAE
BAB83254.1_CsOSC2	cycloartenol	MWCLSR	FVKNSQ	QNVDGGW	QAHDGHW	QRADGSW	QEA-SGGW	DCTGE
XP_019264776.1_NaOSC4	unknown	MWCHCR	FIKASQ	QNVDGGW	QAEDGHW	QLSDGSW	QLQPSGGW	DCTAE
BAA33462.1_PgPNZ1	lanosterol	MWNHCR	FIKISQ	QNVDGGW	QADDGHW	QLPDGSW	QLG-SGGW	DCTAE
NP_001327788.1_AtLSS1	lanosterol	MWCHCR	YIKNTQ	QNKDGGW	QSQDGFW	QLPDGSW	QLC-CGGW	DCTAE
BAE95410.1_LjLAS	lanosterol	MWCHTR	FIKCSQ	QNEDGGW	QAQDGFW	QLADGSW	QLL-SGGW	DCTAE

Supplemental Fig. S1 A motif comparison of OSCs in different species

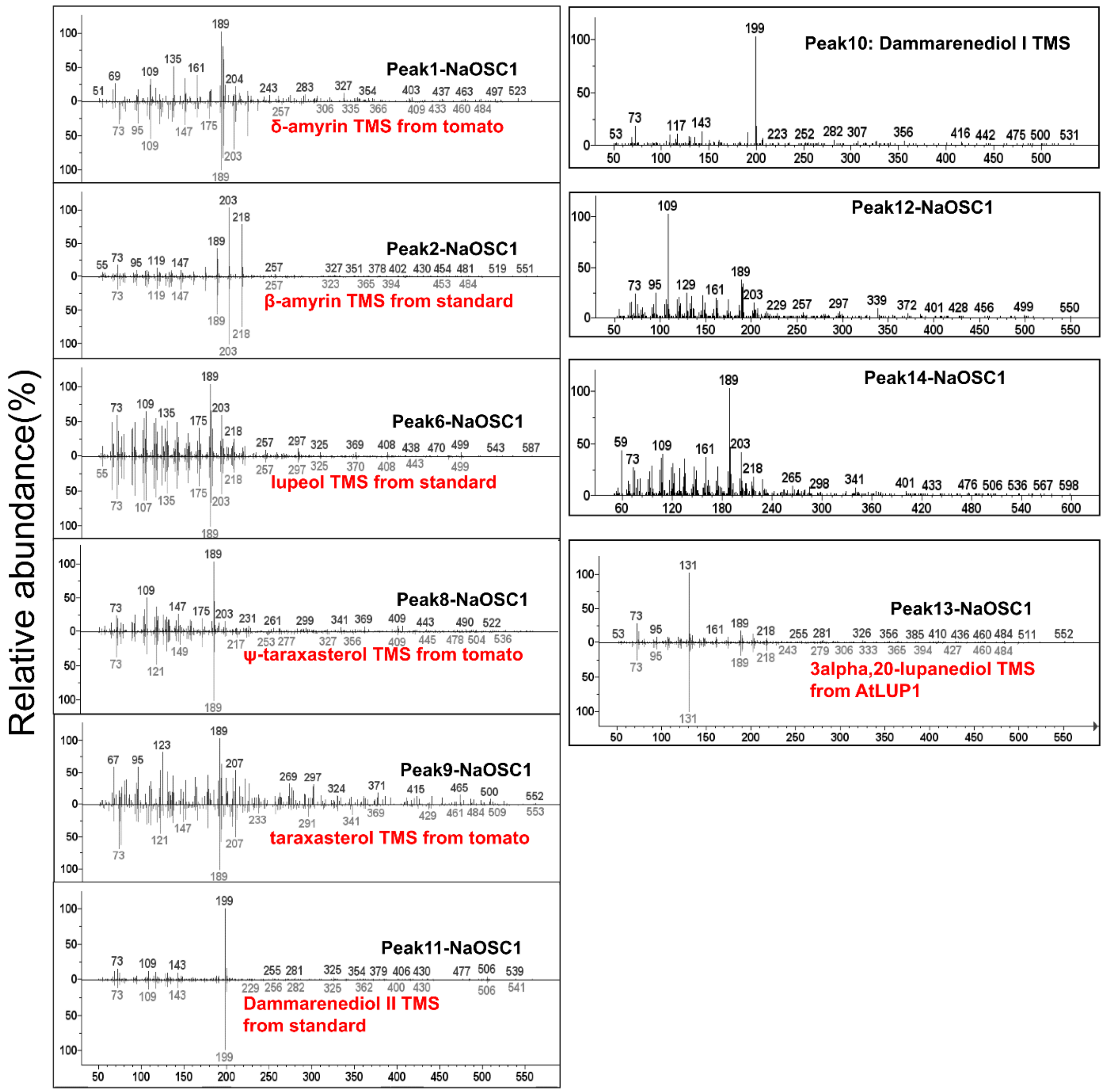

Supplemental Fig. S2 EI mass spectra of the products generated by NaOSC1 or NaOSC2.

TMS: A trimethylsilyl group.

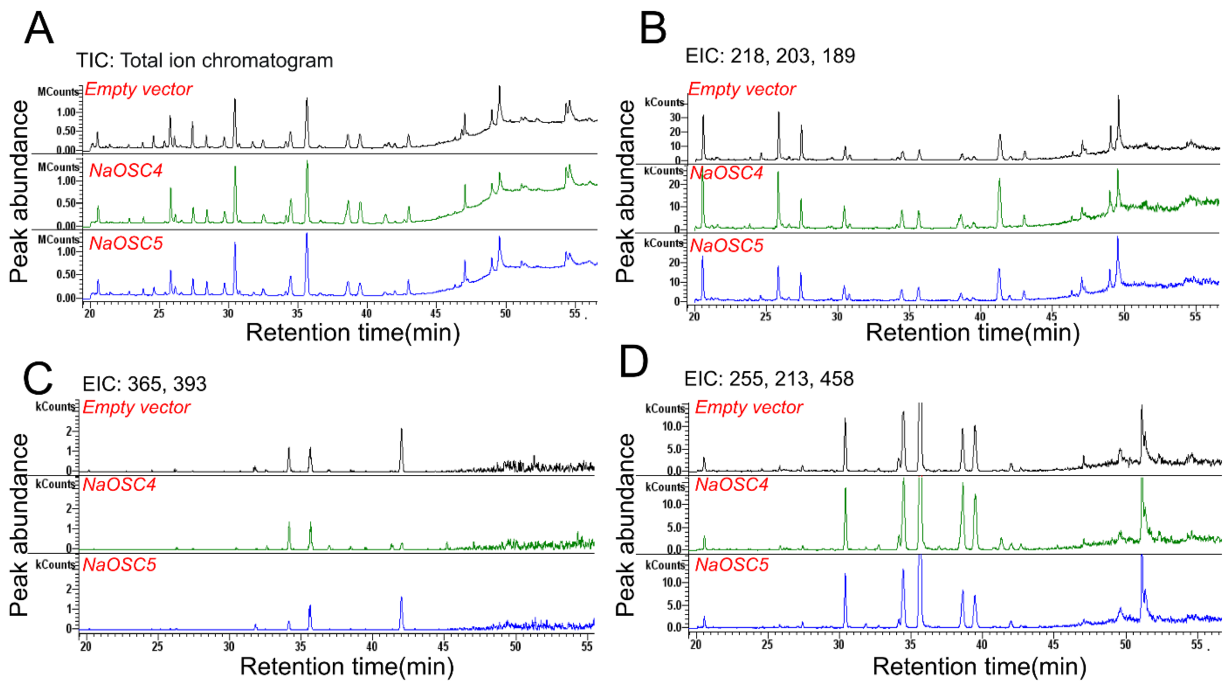

Supplemental Fig. S3 Heterologous expression of NaOSC4 and NaOSC5 in *Nicotiana benthamiana.* **(A)** Total ion chromatogram of tissue from *N. benthamiana* leaves where NaOSC4-5 are transiently expressed. **(B)** Extracted ion chromatogram for specific triterpene fragment ions. **(C)** Extracted ion chromatogram for cycloartenol. **(D)** Extracted ion chromatogram for lanosterol. TIC: total ion chromatogram; EIC: extracted ion chromatogram; 218, 203, 189 are specific m/z for triterpene; 365, 393 are specific m/z for cycloartenol; 255, 213, 458 are specific m/z for lanosterol.

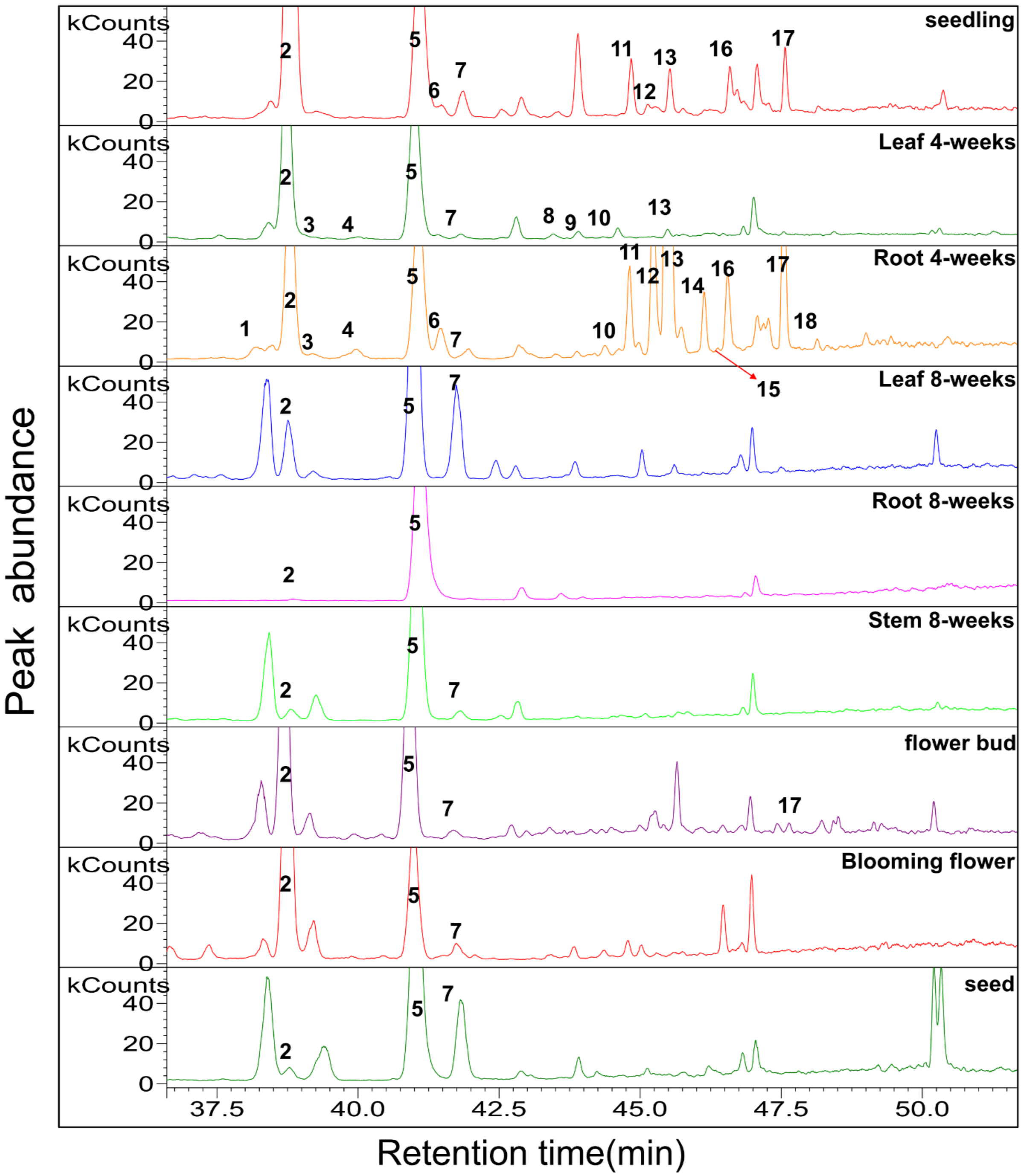

Supplemental Fig. S4 Extracted-ion chromatogram (*m/z* 189, 203,218, 199, 131) of triterpenes in different tissues 1: δ-amyrin; 2: β-amyrin; 3: unknown; 4: germanicol; 5: α-amyrin (internal standard); 6: lupeol; 7: cycloartenol;8: unknown; 9: ψ-taraxasterol; 10: taraxasterol; 11: erythrodiol; 12: dammarenediol I; 13: dammarenediol II; 14: betulin; 15: oleanolic acid; 16: oleanolic aldehyde; 17: betulinic acid; 18: 3alpha,20-lupandiol; 19: unknown.

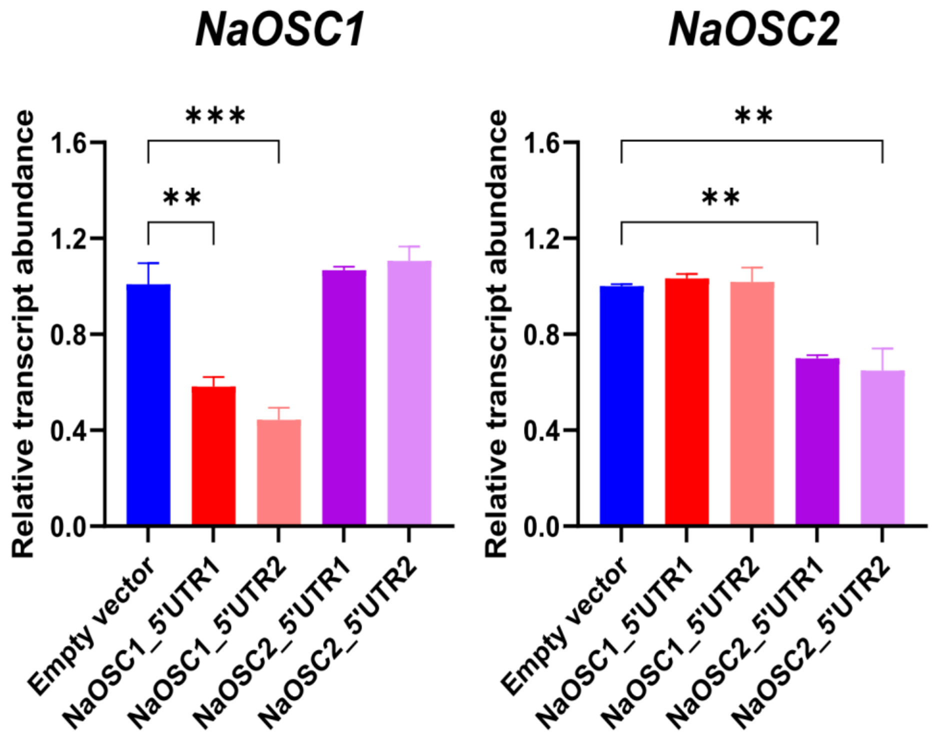

Supplemental Fig. S5 Silencing efficiency of *NaOSC1* and *NaOSC2* in NaOSCs-VIGS plants.

"*NaOSC1_5'UTR1*" and "*NaOSC1_5'UTR2*" represent two distinct VIGS constructs for *NaOSC1*, while "*NaOSC2_5'UTR1*" and "*NaOSC2_5'UTR2*" represent two distinct VIGS constructs for *NaOSC2*. Results of ANOVAs and Tukey's test are shown (mean ± SE, n=3, leaves from four independent plants were mixed together to form one replicate, with a total of three replicates. *, $p \leq 0.05$; **, $p \leq 0.01$; ***, $p \leq 0.001$; ****, $p \leq 0.0001$).

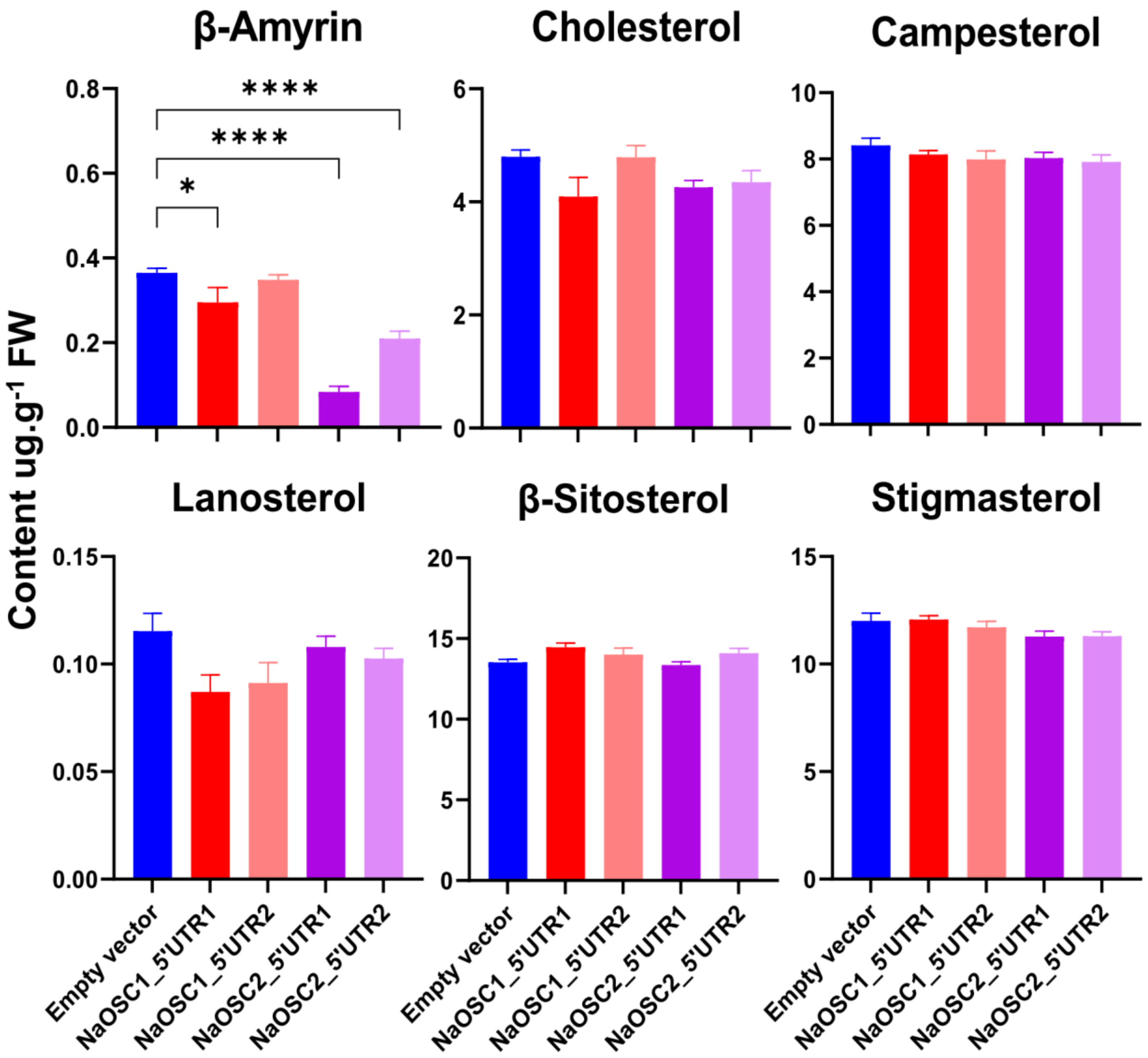

Supplemental Fig. S6 Triterpene and sterol contents in NaOSCs-VIGS -plants. Results of the ANOVAs and Tukey's test are shown (mean ± SE, n=6, leaves from two independent plants were mixed to form one replicate, with a total of six replicates. Results of ANOVAs and Tukey's test are shown (mean ± SE, n=3, *, $p \leq 0.05$; **, $p \leq 0.01$; ***, $p \leq 0.001$; ****, $p \leq 0.0001$).

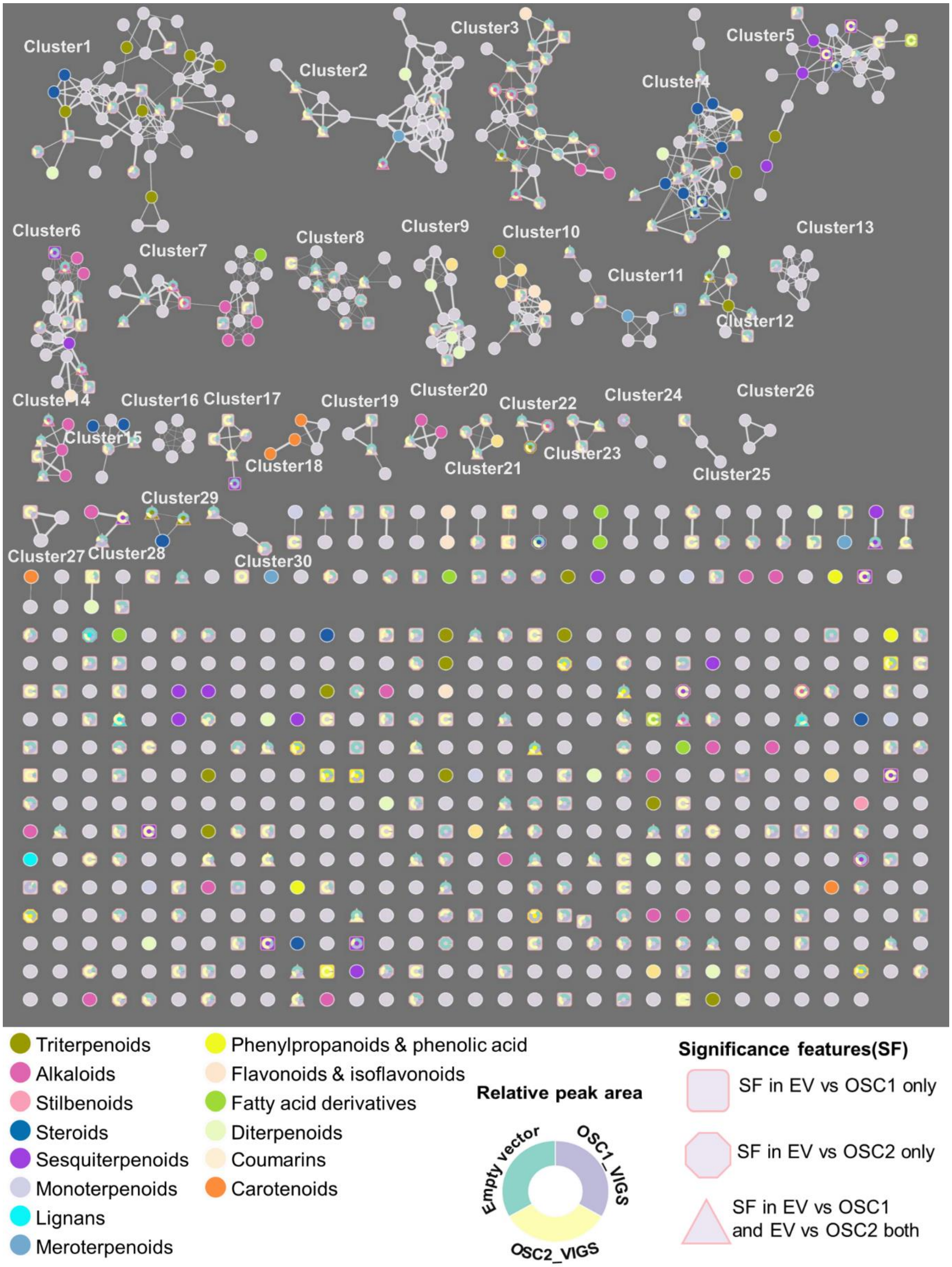

Supplemental Fig. S7 An overview of feature-based molecular networking based on MS/MS data (ESI+). EV: empty vector. SF: the significant feature obtained from Fig. 7

(Wilcoxon rank-sum test, $p \leq 0.05$). The node text indicates the feature ID, while the node color represents the annotated compound type. Square nodes represent features that exhibit significant ((Wilcoxon rank-sum test, $p \leq 0.05$)) changes only when NaOSC1 is silenced compared to the control. Octagonal nodes represent features that exhibit significant differences only when NaOSC2 is silenced. Triangular nodes represent features that exhibit significant changes compared to the control regardless of whether *NaOSC1* or *NaOSC2* is silenced. Tricolor ring nodes represent the relative abundance of features with significant differences in EV (green), NaOSC1_VIGS (yellow), and NaOSC2_VIGS (purple) plants.

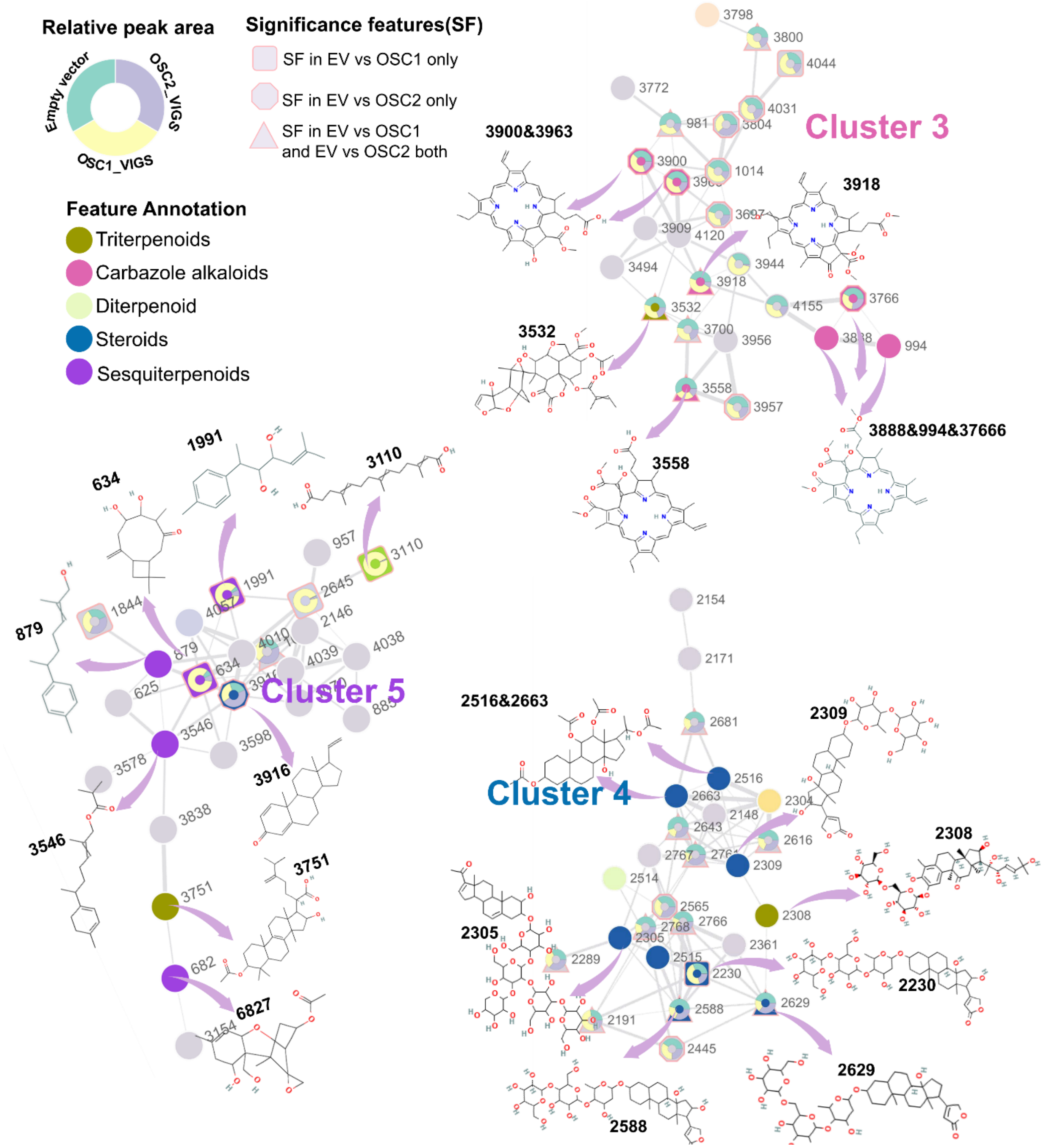

Supplemental Fig. S8 Feature-based molecular networking for carbazole alkaloids, sesquiterpene, and steroids. EV: empty vector. SF: the significant feature obtained from Fig. 7 (Wilcoxon rank-sum test, $p \leq 0.05$). The node text indicates the feature ID, while the node color represents the annotated compound type. Square nodes represent features that exhibit significant changes only when *NaOSC1* is silenced compared to the control (EV: empty vector). Octagonal nodes represent features that exhibit significant differences only when *NaOSC2* is silenced.

Triangular nodes represent features that exhibit significant changes compared to the control (EV) regardless of whether *NaOSC1* or *NaOSC2* is silenced. Tricolor ring nodes represent the relative abundance of features with significant differences in EV (green), NaOSC1_VIGS (yellow), and NaOSC2_VIGS (purple) plants. Pairwise spectral similarity between fragmentation spectra was computed using a modified cosine score. During network construction, edges were filtered to retain those with cosine scores above 0.7 and more than 6 matching peaks. The thicker the edge, the larger the cosine value, and the similarity between the two spectral fragments is about higher.
